# Multiple Spike Time Patterns Occur at Bifurcation Points of Membrane Potential Dynamics

**DOI:** 10.1371/journal.pcbi.1002615

**Published:** 2012-10-18

**Authors:** J. Vincent Toups, Jean-Marc Fellous, Peter J. Thomas, Terrence J. Sejnowski, Paul H. Tiesinga

**Affiliations:** 1 Computational Neurophysics Laboratory, Department of Physics & Astronomy, University of North Carolina, Chapel Hill, North Carolina, United States of America; 2 Psychology Department and Program in Applied Mathematics, University of Arizona, Tucson, Arizona, United States of America; 3 Departments of Mathematics, Biology, and Cognitive Science, Case Western Reserve University, Cleveland, Ohio, United States of America; 4 Department of Neuroscience, Oberlin College, Oberlin, Ohio, United States of America; 5 Howard Hughes Medical Institute, Computational Neurobiology Laboratory, The Salk Institute, La Jolla, California, United States of America; 6 Division of Biological Sciences, University of California at San Diego, La Jolla, California, United States of America; 7 Donders Institute for Brain, Cognition and Behaviour, Radboud University Nijmegen, Nijmegen, The Netherlands; Indiana University, United States of America

## Abstract

The response of a neuron to repeated somatic fluctuating current injections *in vitro* can elicit a reliable and precisely timed sequence of action potentials. The set of responses obtained across trials can also be interpreted as the response of an ensemble of similar neurons receiving the same input, with the precise spike times representing synchronous volleys that would be effective in driving postsynaptic neurons. To study the reproducibility of the output spike times for different conditions that might occur *in vivo*, we somatically injected aperiodic current waveforms into cortical neurons *in vitro* and systematically varied the amplitude and DC offset of the fluctuations. As the amplitude of the fluctuations was increased, reliability increased and the spike times remained stable over a wide range of values. However, at specific values called bifurcation points, large shifts in the spike times were obtained in response to small changes in the stimulus, resulting in multiple spike patterns that were revealed using an unsupervised classification method. Increasing the DC offset, which mimicked an overall increase in network background activity, also revealed bifurcation points and increased the reliability. Furthermore, the spike times shifted earlier with increasing offset. Although the reliability was reduced at bifurcation points, a theoretical analysis showed that the information about the stimulus time course was increased because each of the spike time patterns contained different information about the input.

## Author Summary

Neurons respond with precise spike times to fluctuating current injections, leading to peaks in ensemble firing rate. The structure of these peaks, or *spike events*, provides a compact description of the neural response. We explore the consequences of precise spike times for neural coding *in vivo*, by investigating the spike event structure of virtual cell assemblies constructed *in vitro*. We incorporate diversity of electrophysiological response properties by varying the amplitude and offset of a common stimulus waveform injected *in vitro*. Across multiple trials, spike trains produce precise events in response to upswings in the stimulus, suggesting that such upswings in *in vivo* assemblies may effectively drive postsynaptic targets and transmit information about the stimulus. In simulations and *in vitro*, we identified *bifurcations* in the event structure as the amplitude or the offset was varied. Near bifurcation points, the neural response showed heightened sensitivity to intrinsic neural noise, leading to multiple competing response patterns, and enriching the representation of stimulus features by the ensemble output. The presence of bifurcations could therefore allow an ideal observer to extract more information about the stimulus. Our results suggest that event structure bifurcations may provide a mechanism for boosting information transmission *in vivo*.

## Introduction

Neural recordings *in vivo* are often analyzed with the peristimulus time histogram, which measures increases or decreases in firing rate in response to stimulus onset [1]. Recordings at the sensory periphery, such as the retina and lateral geniculate nucleus (LGN), indicate that spiking responses can be tightly locked to stimulus features with a temporal resolution as high as 1 ms [2], [3], [4], [5]. Ensemble recordings in cortex and hippocampus have shown that populations of cells could dynamically reactivate during sleep and quiet awake periods with high precision [6], [7]. These precisely timed spikes drive target neurons [8], [9], [10], but only a few studies have reported stimulus-locked responses in cortex [11], [12], [13]. The question of how cortical neurons use the information encoded in spike times is fundamental in systems neuroscience [14], [15].

Temporally coherent synaptic inputs due to background cortical activity, uncorrelated with stimulus onset, could reduce the precision of spikes relative to stimulus onset [14]. Thus, whether cortical neurons *in vivo* respond as precisely as those measured *in vitro* depends on the impact of the external stimulus in the context of the background cortical state [14]. We hypothesize that many cortical neurons receive common and/or synchronized inputs [16], because there are 100 times more neurons in the primary visual cortex than there are in the retina or LGN [17], and each spiny stellate cell receives inputs from tens of LGN cells [18], [19], suggesting that the same LGN cell projects onto many cortical cells. We refer to a group of neurons with common input as a neural assembly or ensemble [20], which will be further explained in the Discussion.

**Figure 1:**
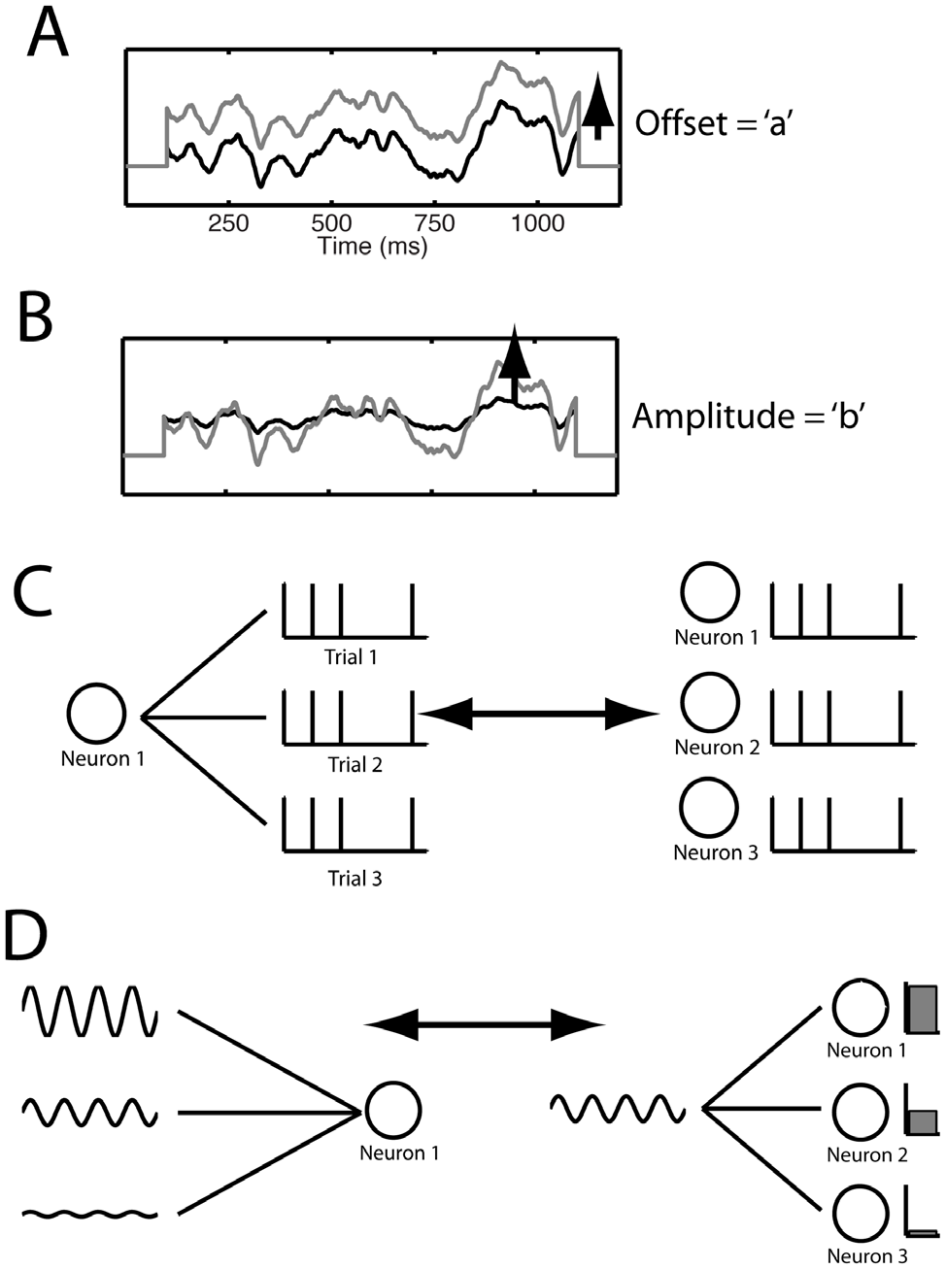
Conceptual foundation for the in vitro experiment. (A,B) Currents with the same temporal waveform are injected multiple times, but either (A) the offset *a* or (B) the amplitude *b* is varied systematically. (C, left) Exactly the same input (including amplitude and offset) is repeatedly injected into the neuron on different trials. (C, right) Under some circumstances the recordings can be interpreted as the response of an ensemble of similar neurons. (D, right) Within an assembly of neurons receiving common input, cells could differ in their membrane properties such as input resistance, level of depolarization, etc. Cells with different input resistances, for instance, would have different gains, represented schematically by bars of different heights. (D, left) The resulting ensemble activity can be approximately reconstructed by repeatedly injecting a common fluctuating current waveform with different amplitude and offset in the same neuron. Hence, the amplitude/offset combinations represent groups of neurons with different intrinsic properties.

We performed *in vitro* experiments to determine how the time-course of neural spike trains is modulated by the strength of an aperiodic current injected into the soma. Although these experiments were conducted *in vitro*, they may shed light on the role of spike timing *in vivo* because *in vitro*, background synaptic activity can be tightly controlled [14], [15], [21], [22], [23], [24], [25]. The synaptic inputs to a neuron *in vivo* can be simulated *in vitro* by injecting aperiodic fluctuating current at the soma. *In vivo*, neurons also receive local cortical recurrent inputs that are modulated by other top-down inputs [26] such as those responsible for covert attention [27], [28]. We approximated these effects *in vitro* by changing the amplitude and offset of the stimulus waveform (Figure 1A and B).

The response of one neuron obtained across multiple trials for different amplitudes or offsets can be interpreted as the response of a neural ensemble under the assumption that the neurons in the ensemble do not strongly interact with each other (Figure 1C and D). According to this interpretation, precise spike times of a single neuron measured across trials can also be understood as synchronous volleys of a neural ensemble [22], [29], which are effective in driving postsynaptic cells both *in vivo* [30] and *in vitro* [31]. The strength of a volley depends both on the number of cells in the ensemble that produce a spike, which is related to the reliability across trials, and on their degree of synchrony, which is related to the precision. In a real neural ensemble, the interactions between the neurons will affect both the number of cells emitting a spike and their amount of synchrony, but the independence assumption [32] provides a starting point for thinking about how a stimulus can be represented across a population of similar neurons.

Reliability is in principle different from precision [14]. We developed an event-based analysis, for which the sets of spike trains are represented as a set of spike time events. This analysis can be performed for spike trains recorded from the same neuron across trials as well as for spike trains of different neurons recorded on the same trial, as in ensemble reactivation in cortex or hippocampus. For ease of presentation, we will describe the analysis in terms of trials. Spike time occurrences, called *events*, are temporally localized concentrations of spike time density across trials, which can be characterized in terms of their occurrence time, precision and reliability. We investigated how these quantities vary with stimulus amplitude and offset. In the following we will use precision and jitter interchangeably to refer to the temporal resolution of spike times.

We report three key results: First, we found that spike trains change with stimulus amplitude in such a way that the information about the stimulus time course was preserved. Some information about the amplitude is only reflected in the trial-to-trial variability and thus needs to be reconstructed based either on multiple trials or multiple units operating in parallel. Second, the general behavior as a function of drive parameter (amplitude or offset current) could be characterized in terms of spike patterns and bifurcation points in the dynamics of the membrane potential. Spike patterns are within-trial spike correlations, which may be due to afterhyperpolarization currents [33] and other slower currents activated by action potentials. At bifurcation points the spike times changed rapidly in response to a small change in parameter value; this change resulted in multiple spike patterns. The number of different spike patterns provided important information about the stimulus time course. We found that this number was highest for the intermediate amplitudes used in our experiments, as a consequence of the presence of bifurcation points. Third, we used an event-based analysis, which made it possible to semi-automatically analyze spike train data [34]. The entire procedure is characterized by four parameters, for which heuristics are given [34]. The main advantage of the event-based analysis is that it does not rely on fitting a specific parametric model for the neural dynamics based on the stimulus [35], [36],[37],[38]; rather it models the data directly.

Preliminary reports have appeared in abstracts [39], [40], [41], [42].

## Results

### Spike pattern sensitivity to stimulus amplitude and offset

We define the stimulus amplitude *β* and DC offset *α* as follows: Given a fixed, fluctuating aperiodic current waveform, *h(t)* with a duration of 1000 ms, constructed to have an average value of zero and a given variance, we inject a current stimulus of the form *I(t) = α+β h(t)* (Figure 1A and B). The mean DC current injected is *α* and the root mean square (RMS) size of the fluctuation is *β* times the RMS size of *h*. On any given set of trials we predetermined a set of relative amplitudes *b* and offsets *a*, which we scale with a multiplicative factor ν. This factor is determined on a cell-by-cell basis in order to adjust each suite of stimuli to the cell's intrinsic firing properties (as described below). The resulting stimulus is *I(t) = ν (a+b h(t))*, so the stimulus offset is given by *α = ν a*, and the stimulus amplitude is given by *β = ν b*. As the quantities *a* and *b* are common across experiments, the results will be presented in terms of *a* and *b* (the latter as a percentage) rather than α and *β*.

The current so constructed was injected into the soma of layer 5 pyramidal cells in a slice of rat prefrontal/infralimbic cortex. The fluctuating drive was the same on each trial, but for the first experiment we used eleven different amplitudes, expressed as percentages. The first step was to determine the scaling factor ν, which varied from neuron to neuron according to its intrinsic properties. We used between 18 and 51 trials per amplitude and performed experiments on 10 different cells. For 8/10 cells, *b* ranged from 0% to 100% in steps of 10%, whereas for 2/10 cells *b* was 20% to 100% in steps of 8%. Because the injected waveform was prepared off-line and stored in a file, at the time of recording we could only adapt the overall gain *ν* to the properties of each neuron. The overall gain (*ν, taking values* between 0.4 and 5) was chosen such that the neuron produced at least one spike for the lowest amplitude (*b = 0 or 20%*), which was achieved for 8/10 cells.

**Figure 2:**
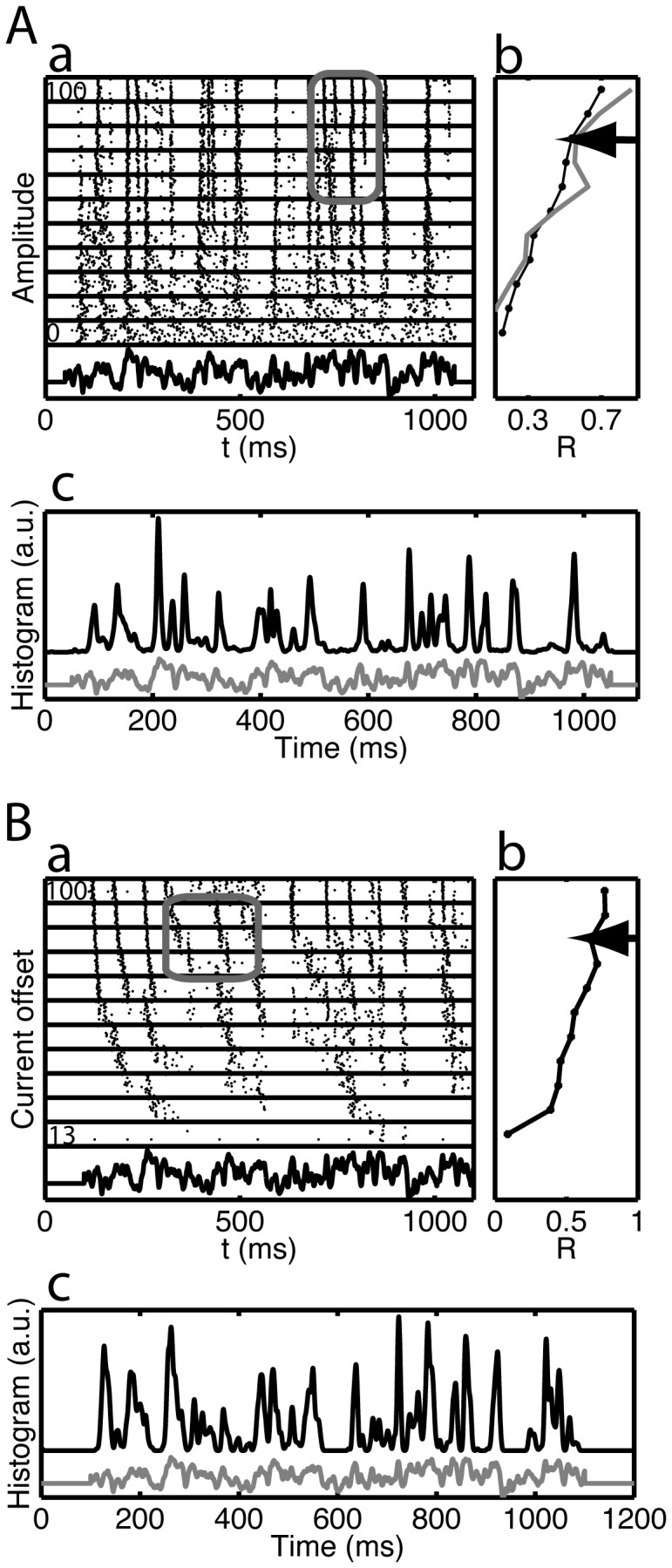
Spike timing in response to a fluctuating current is robust against changes in amplitude and offset. Responses of two Layer 5 pyramidal cells in a slice preparation of rat prefrontal cortex. In (A) the amplitude of the fluctuating current was varied, whereas in another cell (B) the current offset was varied. For each panel: (a) the rastergram, (b) the R-reliability (Schreiber measure with sigma = 3 ms, see Methods) versus amplitude or current offset and (c) the average spike time histogram across all values of either the amplitude or offset. In subpanel (Ab), the gray curve is the R-reliability based on only spikes in the time interval between 700 ms and 900 ms. The stimulus waveform is shown for reference at the bottom of subpanels (a) and (c). Each line in the rastergram represents a spike train obtained on a trial, with the ordinate of each tick representing a spike time. The spike trains are ordered in blocks (delineated by horizontal lines) based on the amplitude or offset of the injected current, expressed as a percentage, with the highest amplitude or offset on top. In (A) the amplitude ranges from 0% to 100% of maximum amplitude. In (B) the current offset ranges from 0.05 nA to 0.3 nA; indicated as a percentage (0.05/0.3 = 13% of maximum; 0.3/0.3 = 100% of maximum). Within each block the trials are in the order they were recorded, with the earliest trial at the bottom. The arrows in subpanels (b) indicates the dip in the R-reliability, which is related to the spike train dynamics highlighted by the corresponding gray box in subpanels (a). This behavior is related to the presence of so-called bifurcation points.

Rastergrams for one representative cell are shown in Figure 2A, in response to an injected current waveform. A spike-time histogram for all values of the current step and scaling factor are shown in the bottom panel. The rastergram consists of blocks of constant amplitude, with the highest amplitude on top. Within each block the trials are in the order they were collected, with the earliest at the bottom. In the rastergram (Figure 2Aa), events are visible as spike alignments that appear for low to intermediate amplitudes and that become sharper as the amplitude is increased. Overall this graph suggests that both the precision, the jitter in the spike times belonging to an event, and the reliability, the fraction of trials on which a spike is present in an event, improve with amplitude. The trial-to-trial variability was characterized using the *R*-reliability (see Methods) with a sigma value of 3 ms [43]; that is, spikes in two different trials with a time difference of less than 3 ms are considered effectively coincident. This measure does not distinguish between changes in reliability and changes in precision (as demonstrated previously [14], [34]), which would require an event-based analysis. Overall, *R* increased with amplitude (Figure 2Ab), but there were dips, indicated by the arrow. As will be shown below, the presence of a dip is characteristic of a bifurcation point, where a small change in the stimulus can lead to a large shift in the pattern of spikes.

The spike times for an event shifted only slightly as a function of stimulus amplitude, as demonstrated by the relatively sharp peaks in the spike time histogram across all amplitudes (Figure 2Ac). The distribution of spikes was broad, reflecting the responses for the lowest amplitudes, which were not strongly stimulus-locked, but had a sharp, approximately symmetric peak, corresponding to the highest amplitudes. To interpret these results, consider a neural ensemble comprised of different neurons receiving feedforward inputs with strengths that are different because the synaptic inputs could have different decay time constants or short-term plasticity [44] and the neurons could have different input resistances and resting membrane potentials [45]. The *in vitro* experiments show that despite this diversity the ensemble would produce volleys that are effective in driving postsynaptic neurons and transmitting information about the time course of the input [21]. This effect was also observed *in vivo* comparing the responses of different cat LGN neurons to the same flickering light stimulus [4]. Despite a wide range of firing rates, some of the events in these recordings were consistent across neurons of the same type even in different cats.

For the second set of experiments, the current offset, *a*, was varied between 0.05 and 0.3 nA in 10 equal steps, while the amplitude of the stimulus waveform was held constant at b = 0.05 nA (for the example in Figure 2B, in which amplitude is expressed as a percentage). We presented 7 such stimulus sets (amplitudes b = 0.02 to 0.06 nA) to 5 different cells and recorded the responses on 17 to 36 trials, with overall gain factors *ν* between 2.7 and 3. The overall behavior was similar: Precise spiking was obtained at all current offsets, with some common event times (Figure 2Ba and c), and the reliability measure R increased with current offset (Figure 2Bb) and also displayed a dip (arrow in Figure 2Bb). The differences between the effects of varying offset and varying amplitude were, first, the firing rate increased when current offset was varied (from 1.0±1.4 to 15.5±0.8 Hz for Figure 2B, mean ± standard deviation across trials, corresponding to an increase from the lowest to the highest offset, relative to the maximum rate, of 0.92; across the population, N = 9, this was: mean 0.88, range 0.32 to 1.0) more than for the case where the amplitude was varied (from 10.5±1.1 to 14.5±0.9 Hz for Figure 2A, corresponding to 0.28, across the population, N = 6, mean 0.75, range 0.28–1.0). This is because the overall level of depolarization increased, whereas for increasing amplitude not only did the peaks increase, but the troughs also got deeper, which meant that some spikes would appear and other spikes would disappear. Because we could not perfectly adapt the relative level of amplitude and offset, such that for zero amplitude there already was some spiking, the across-the-population difference in firing rate between the two cases was smaller. Second, for a nonzero firing rate, the neurons immediately phase locked to the waveform in the current offset case, which resulted in higher reliability (we excluded the first current offset, for which the neurons only spiked a few times), even at a rate of a few Hz, compared to the amplitude case. Third, the distribution of spike times in an event was asymmetric, with the peak skewed to earlier times. This was because the increasing depolarization made the spikes reach threshold earlier. Note, however, that for the low current offset trials the first spike times appeared to drift with current offset, but they actually shifted to earlier events on some trials.

### Event based analysis for pattern identification

**Figure 3:**
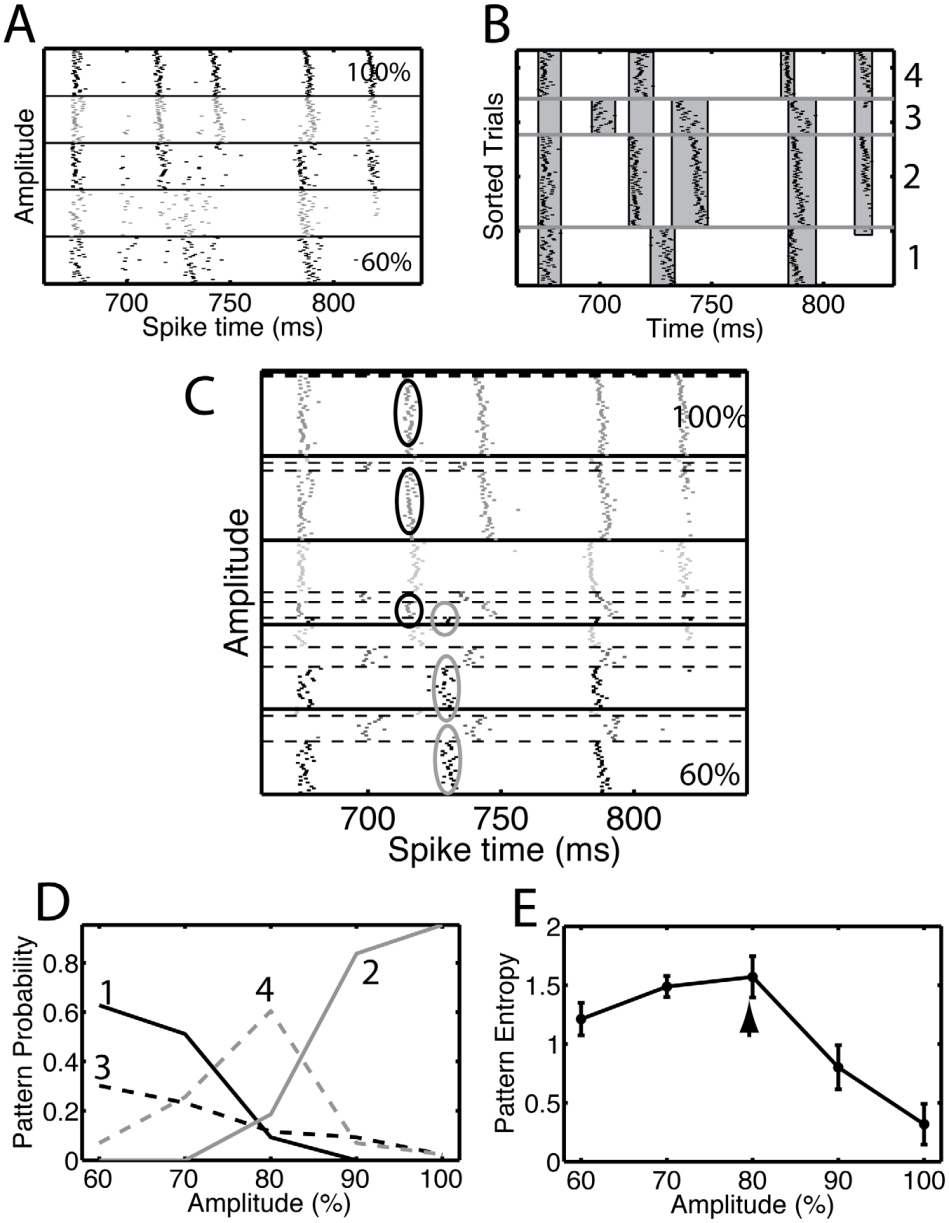
Bifurcation points led to multiple spike patterns that persisted across multiple amplitudes. (A) The rastergram for the data shown in Figure 2A for amplitudes between 60% and 100% and during the time segment between 650 ms and 850 ms. (B) The analysis procedure suggested that there were four clusters, each corresponding to a spike pattern. We show the rastergram with the trials sorted according to their cluster membership. The numbers on the right side are the cluster index. The gray vertical bands show the detected events that remained after applying a procedure to merge events common to multiple clusters. We used the value *t_ISI_* = 3 ms to detect the events using the interval method and the value *t_ROC_* = 0.50 to find and merge common events. (C) Rastergram of the clustered data shown in panel A. Each block (separated by thick black lines) corresponds to a different amplitude, with the lowest amplitude at the bottom and the highest amplitude at the top. Within each block, the trials are ordered based on their cluster membership. The clusters are separated by thin dashed lines. Two events are highlighted: the ones in the black ellipses, whose reliability increased with amplitude and the ones in the gray ellipses, whose reliability decreased with amplitude. (D) The pattern occupation (or probability) for a given amplitude is the fraction of trials on which that pattern is obtained. We show the pattern occupation as a function of amplitude for the four patterns that were detected, as indicated by the numbers in the graph. (E) The diversity of patterns observed for a given value of the amplitude is quantified as the entropy of the pattern distribution. The entropy as a function of amplitude has a peak at 80% (arrow), indicating that the pattern diversity is largest for that amplitude. The error bars represent the standard deviation of the entropy determined using a resampling procedure (see Methods). There is no correction for the bias, which took values between 0.02 and 0.05 bits.

Identification of events in a peristimulus time histogram is essential for the analysis of stimulus encoding *via* spike time patterns. Because the events visible in the multi-amplitude rastergrams (Figure 2) persist across amplitudes, the precision, reliability and mean spike time of events can be compared across amplitudes (see Methods). One strategy for event-based analysis would be to find events for each amplitude and merge events common across amplitudes. In Figure 3 we show the results of an alternative strategy, in which spike train ensembles generated by the five highest amplitudes are analyzed at the same time. With this approach the underlying pattern-finding procedure (see Methods) is more robust because there are more trials in each pattern [34]. Our analysis revealed the presence of four patterns (Figure 3B), which led to 8 events, some of which were common to multiple patterns. We recall that *events* are temporally localized concentrations of spike time density across trials and *patterns* are transient multiplicity of spike response. In panel B the patterns are divided by gray horizontal lines, whereas all the spikes belonging to an event are enclosed in a gray vertical box.

Because there were more amplitudes (five) in the data set than there were patterns (four), a given pattern had to persist across multiple amplitudes. We plotted the rastergram in blocks of constant amplitude, and sorted the trials on the basis of the pattern they expressed (Figure 3C). The fraction of patterns that were present varied across amplitude and was quantified in Figure 3D. As the fraction of trials with the second pattern increased (Figure 3D, line 2), the fraction of trials on which the first pattern was present decreased (Figure 3D, line 1). Hence, the reliability of events in the second pattern increased (black ellipses in Figure 3C), whereas the reliability of events in the first pattern decreased (gray ellipses in Figure 3C). As patterns fade in and fade out during variation of amplitude, the mix of patterns present across trials at a given amplitude varies with amplitude. Thus the non-monotonic change in reliability with amplitude indicated by the arrows in Figure 2Bb reflects the changes in the mix of patterns.

The diversity of patterns present for a given amplitude is quantified using the entropy of the pattern distribution (Figure 3E). For these data, the entropy was maximal at a specific amplitude (see Methods, arrow in Figure 3E). In other data sets and segments, the entropy decreased monotonically with increasing amplitude because for higher amplitudes only one pattern survived. These results are relevant to the amount of information that can be extracted about the temporal dynamics of the injected current from the spike time patterns and is discussed below.

An important question is what aspects of the membrane potential are reflected in, or can be reconstructed from, the measured spike times, because the membrane potential itself is not accessible from *in vivo* extracellular recordings. Without the membrane potential little information can be obtained about excitatory and inhibitory inputs to the neuron [46], needed to test hypotheses about computational mechanisms. To address this *in vitro*, we obtained multiple trials and analyzed recordings where the same waveform with the same amplitude was injected on each trial. For these experiments, the fluctuating waveform was extended to 1700 ms and was preceded by a constant current offset of 200 ms (in addition to 50 ms zero current at the start of all current injections). In these experiments, the initial current offset took eleven different values, the influence of which are discussed below. We injected this drive in nine experiments using eight cells, with between 10 and 35 trials each (110 to 385 trials overall, ignoring the initial current offset) and an overall gain with values between ν = 0.9 and 4.7. The main difference between this data set and the ones used in Figure 2 and 3 is that there are more trials available for statistical analysis.

**Figure 4:**
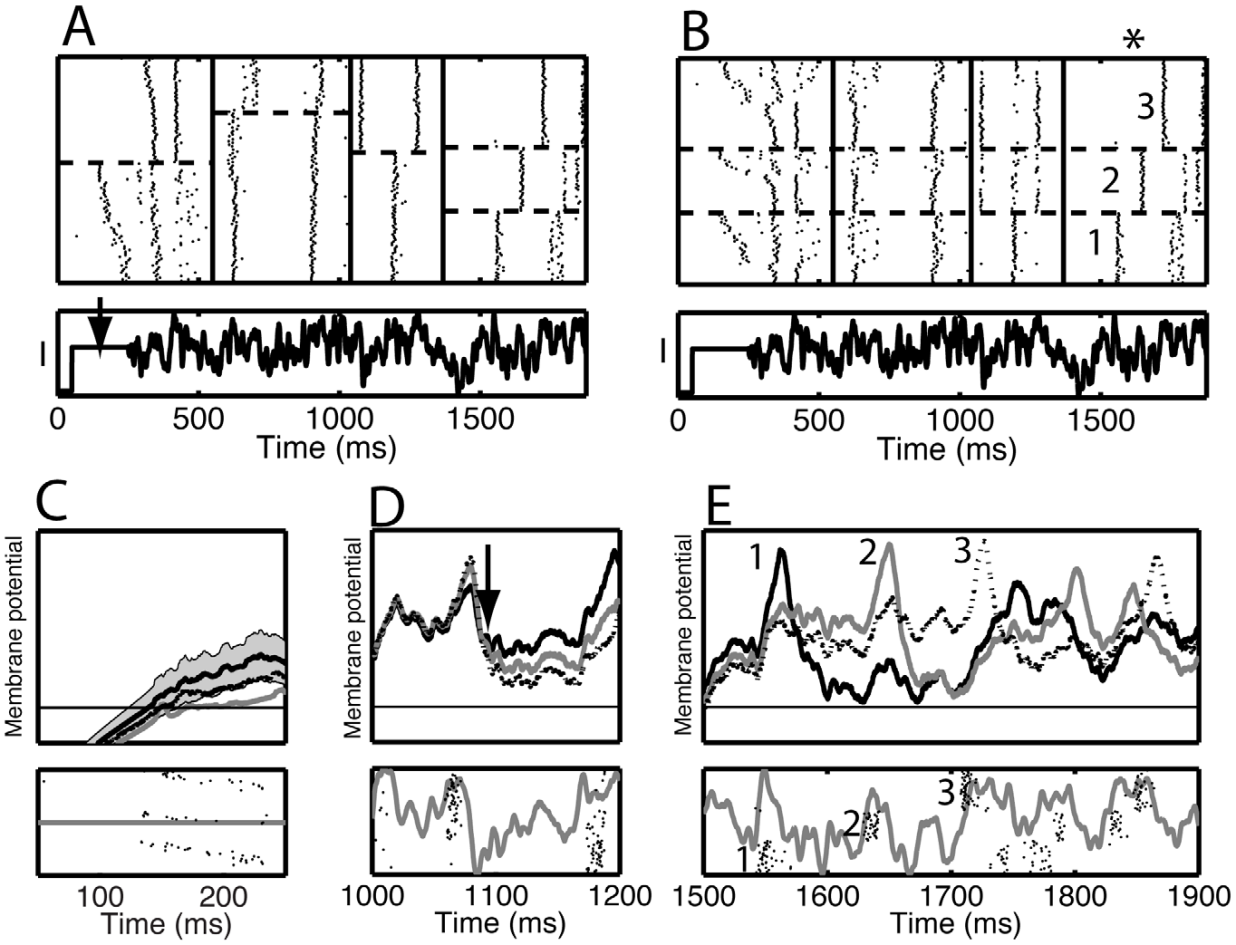
Spike patterns corresponded to voltage patterns. The single-amplitude data set was divided into four time segments. (A) Segment-by-segment rastergrams. In each segment trials were ordered according to the cluster membership in that segment. The clusters are separated by horizontal dashed lines, whereas segments are indicated by vertical lines. Because the trial order varied from segment to segment, spikes on the same row but in different segments are not necessarily obtained in the same trial. There was a 200 ms long constant current step (arrow), whose amplitude took eleven different values (only one is shown). (B) Rastergram with trials in each segment ordered based on their cluster membership on the fourth segment (asterisk) – each row thus represents the same trial across all the segments. At the bottom of A and B the current waveform is repeated for reference. (C–E) The analysis procedure found 3 spike patterns in the fourth segment (between 1500 ms and 1900 ms), labeled 1 (solid black curve), 2 (dotted black curve), and 3 (gray curve). In each of the panels C–E, we show (top) the voltage traces averaged across all trials expressing that pattern (the y-axis covers the range from −65 to −35 mV) and (bottom) the current waveform (gray curve) together with a rastergram where the trials were ordered based on the cluster membership in the fourth segment. The spikes were shifted to the left by 12 ms so that they were approximately aligned with an upswing in the injected current. In (C) the gray bands indicate the plus or minus two standard error range for the black curve. The arrow in (D) indicates the point where differences between the voltage traces appeared. Note that the spike patterns are clearly visible in panel E, but that they are hardly visible in panel D indicating a low correlation between patterns expressed in different segments. Trials are sorted in the same order in panels C–E.

Spike patterns correspond to within-trial correlation between spike times. We determined how long these correlations persisted by applying the spike pattern analysis to approximately 500 ms long segments from seven of the nine available data sets from which we show one (Figure 4A). The segment length was chosen such that at least two events, and no more than six, were present. Within each segment the trials were ordered according to the pattern they expressed in that segment. This shuffles the trials differently in each of the segments and the spikes on a single row of the rastergram most likely correspond to a sequence of segments from different trials. We then determined how well the pattern expressed on a trial during one time segment predicted which pattern was expressed in a preceding or following segment, that is, the between-segment correlation of the patterns that neurons express. Strong correlation means that if in one segment a group of trials express the same pattern, they will also do so in the other segment. In Figure 4B, the trials were reordered in each segment based on the order in the last (fourth) segment (indicated by the asterisk). For this case, each row is one and the same trial on each segment in contrast to the display in panel A. This panel shows that even though a group of trials expressed the same pattern during segment 4, that same group expressed a mixture of patterns during segment 3 – indicative of a low between-segment correlation. This association between different segments is best expressed as the normalized mutual information between the pattern classification of a trial in two segments (I_N_, see Methods, which is primarily used here to summarize a two-dimensional array of transfer probabilities rather than to make statements about information content). The maximum value was normalized to one, which occurs if the classifications are identical. The I_N_ (bias; std) between the classification in segment 4 and that in segments 3, 2 or 1 was 0.20 (0.01; 0.04), 0.003 (0.009; 0.012), or 0.025 (0.007; 0.020), respectively.

We further analyzed the three patterns uncovered in segment 4 between 1500 and 1900 ms. For each pattern, the voltage traces were averaged across all corresponding trials and the standard deviation was used as an estimate for the trial-to-trial variability. The mean voltage traces differed not only because the neurons spiked at different times, but also because the conductances associated with the afterhyperpolarization following the spike had a long-lasting influence on the response to the current injection (Figure 4E, top; averages from patterns 1 to 3). The spikes reflected periods where the injected current had a large positive slope, but each pattern was triggered by a different subset of these upswings (Figure 4E, bottom). Once a spike was produced, the neuron did not spike during an otherwise viable upswing shortly thereafter, even though it had produced a spike there on other trials during which it expressed an alternative pattern. For instance (Figure 4E, bottom), on trials labeled 1, the neuron did not spike in response to the upswing that caused the neuron to spike on trials labeled 2 because the membrane was hyperpolarized.

To uncover correlations that persisted across segments, for each pattern expressed on segment 4, we averaged the voltage traces across all trials belonging to that pattern for the entire duration of the trial. For clarity, we only show three time intervals (Figure 4C to E). In the first interval between 100 and 250 ms there was a small difference in the mean membrane potential (Figure 4C), which had disappeared by t = 1000 ms (Figure 4D), but reappeared after a deep hyperpolarization (Figure 4D, arrow). This difference then led to three clearly distinct voltage patterns in the last interval (Figure 4E). Thus, whether or not a neuron spiked at a given transient depolarization determined the subsequent firing pattern for hundreds of milliseconds, a time scale comparable to the time needed to process a visual image.

The neuron's internal state determined whether or not a neuron fired a spike at a given time, which depended on its previous history as well as the current membrane potential. This could include the height of the depolarizing step that preceded the fluctuating current, or whether the trial was at the beginning or end of the experiment. The normalized mutual information between the trial number and the pattern on segment 4 (the degree of non-stationarity) was I_N_ = 0.059 (bias: 0.001; std: 0.011), and between the height of the offset and the pattern it was I_N_ = 0.050 (bias: 0.006; std: 0.013). This analysis shows that there was an influence but only a small fraction of the variability can be explained by these two factors.

Among the other eight data sets, two had too low firing rates, which meant that there was less than one spike during segment 4, these were not further analyzed. For the remaining six data sets (see Figure S1), we found that in five of them non-stationarity caused high normalized mutual information between the trial number and the resulting pattern on segment 4, which also led to large values for the I_N_ between patterns on different segments. The one remaining data set was stationary (052701; Figure S1C), but, as assessed by the normalized mutual information, there was little correlation between patterns on different segments. Our procedure was robust against non-stationarity in the sense that for small shifts in the spike times during the course of the experiment, these spikes would still be assigned to the correct event, whereas for large shifts, the later trials would be classified as a different pattern. Nevertheless, the typical procedure was to remove the last few trials from a non-stationary data set to obtain an approximately stationary one to analyze.

Apart from suggesting a mechanism for how intrinsic neural properties generated correlations between patterns in different segments, we also showed that the analysis procedure was robust against the effects of non-stationarity

### Bifurcation point analysis of spike patterns

**Figure 5:**
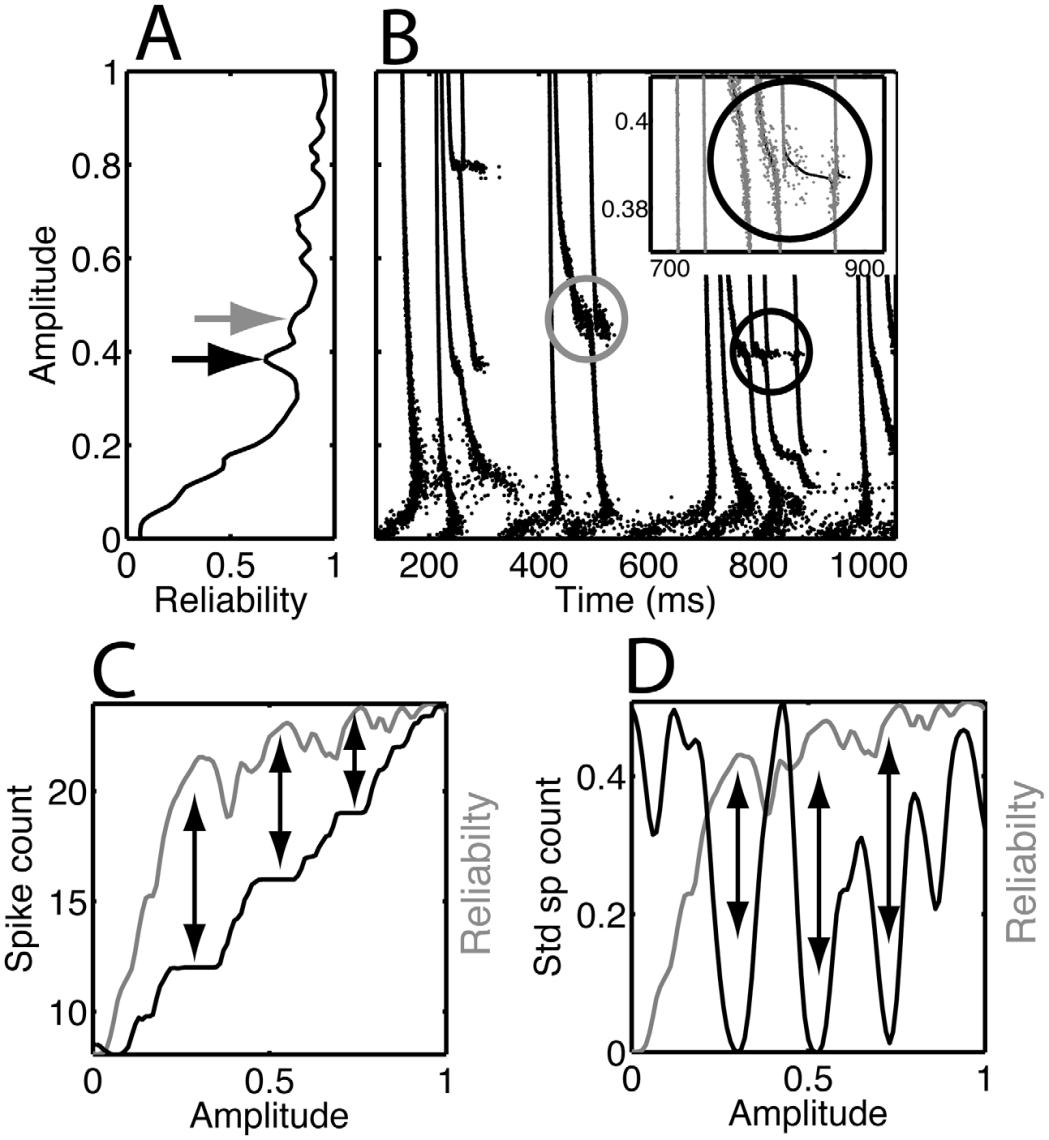
Bifurcation points were observed in model simulations for amplitudes at which the spike count changed. We show the (A) R-reliability (sigma = 1 ms) and (B) rastergram as a function of amplitude obtained from simulations of the Wang-Buzsaki model neuron [22], [47], using the same injected current as in the recordings from neurons. The dip in reliability indicated by the black and gray arrow in (A) corresponds to the bifurcation in the black and gray circle in (B), respectively. The inset in B is the close up of the rastergram shown in the black circle. We plot (C) mean spike count and (D) standard deviation of the spike count across trials versus the amplitude. The gray curve is the R-reliability replotted from panel A, the full range for R, 0 to 1, is represented in the graph. Peaks in the reliability, indicated by the double-headed arrows, correspond to (C) plateaus in the spike count, for which (D) the trial-to-trial variability in the spike count was small.

Without knowing the internal state of a neuron it is difficult to draw any conclusions about dynamical mechanisms that might be responsible for the observed diversity of spike patterns. The same current inputs were used to study the Wang-Buzsaki (WB) model neuron [47] modified by including a small additive noise current as a source of independent trial-to-trial variability (see Methods). We simulated 50 trials for each of 101 different amplitudes *b* between 0 and 100% (the stimulus *h(t)* was normalized to have zero mean and unit standard deviation). Figure 5A shows the reliability curve, which was smoothed by a three-point running average, and Figure 5B shows the corresponding rastergram with matching amplitudes. For clarity we only displayed half of the trials and half of the amplitude values in the rastergram.

The R-reliability generally increased with amplitude but showed multiple local minima. Two such minima are highlighted by the arrows and correspond to the spike train features inside the circle of matching gray-scale in Figure 5B. The bottom half of the gray circle intersects two reliable spike times and at higher amplitudes the top half of the circle intersects three spike times. The spike train ensemble exhibited a bifurcation within this region because one pattern of events branched into another. For continuous models (Hodgkin-Huxley, as opposed to the leaky integrate-and-fire) the spike time “bends”, that is, shifts to earlier times, after which it stops shifting because it arrived at a previously-subthreshold peak, and at some point the original spike time (the one that was shifted) emerges again. For the leaky integrate-and-fire model, new spike times get inserted *de novo*. Moreover, dynamics changed rapidly for small changes in the parameter value. Inside the circle there were fuzzy clouds of spikes corresponding to multiple competing patterns for a given amplitude. As the amplitude increased, the fraction of patterns with two spikes decreased, whereas the fraction of those with three spikes increased. A similar transformation took place within the black circle, where a three spike-time pattern occurred on the bottom half of the circle and as *b* increased a four spike-time pattern appeared on the top half of the circle.

The peaks in the reliability (gray curve) coincided with plateaus where the spike count was constant as a function of stimulus amplitude *b* (black curve) (Figure 5C), and the variability of the spike count was minimal (Figure 5D). A similar behavior was obtained when the current offset *a* was varied, holding the amplitude *b* fixed. In both the model and in the *in vitro* experiments, the firing rate increased more rapidly with increasing normalized offset (*0≤a≤100%*) than with normalized amplitude (*0≤b≤100%*); and the reliability was high even at low firing rates. In contrast, for small amplitudes, a low R-reliability similar to that in response to a current step was obtained, because the stimulus lacked temporal structure.

### Ensemble representation of stimulus at a bifurcation point

The spike train ensemble represents specific features of the stimulus with the timing, reliability and precision of spike event patterns in the ensemble. Away from bifurcation points, there is only one spike pattern and the neuron spikes with a high precision and reliability at a subset of stimulus upswings. When there are more upswings than there are spikes, information about the time course of the stimulus is lost. By contrast, near a bifurcation point the dynamics is more sensitive to noise, and multiple spike patterns are obtained with non-overlapping event times. Each pattern provides information about a subset of stimulus features. Given a natural (fluctuating) input, the precisely timed spikes provide evidence for an upswing in the stimulus. Absence of a spike could reflect a downswing of the stimulus or just the relative refractoriness of the cell following a spike. The coexistence of multiple patterns means the spike train ensemble provides a richer description of the stimulus and is therefore in principle more informative, in the sense that an ideal observer can extract more information about the stimulus [48].

**Figure 6:**
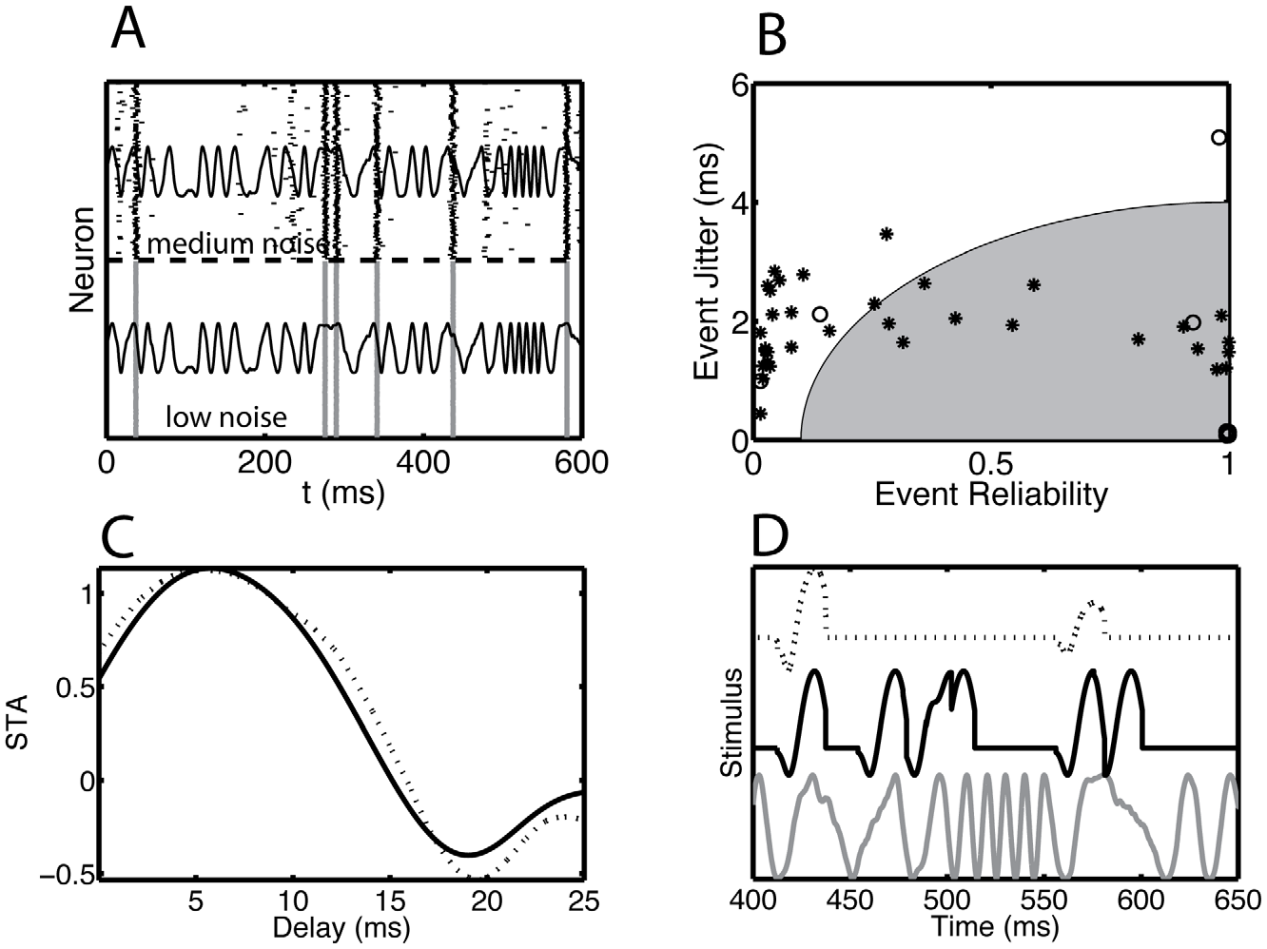
Information about the time course of the stimulus waveform is increased at bifurcation points because of the presence of multiple spike patterns. Data from an example model neuron as described in Figure 5. (A) Rastergram for a short time segment across 100 trials for a (bottom) low-noise and (top) medium-noise model neuron. The noise level refers to the magnitude of a white noise current that varied from trial-to-trial relative to the amplitude of the repeated fluctuating current waveform (shown as a thin solid line on top of each rastergram). For low noise, the neuron spiked only at six events, whereas for medium noise there were additional events. (B) We calculated the reliability and jitter for each event for the entire stimulus duration (1100 ms). The open circles represent the low-noise, and the asterisks represent the medium-noise result. The gray-filled region schematically represents the combination of jitter and reliability for which a putative postsynaptic neuron would generate a spike. (C) The spike-triggered average obtained across the entire stimulus period for (solid line) the medium-noise neuron and (dotted line) the low-noise neuron. (D) The stimulus waveform reconstructed using the low-noise (dotted line) and medium-noise (solid line) spike trains was compared to the actual stimulus waveform (gray solid line). We used an event-based reconstruction, where each extracted event contributed equally to the reconstruction regardless of reliability and jitter, as long as the reliability exceeded 5%. The three curves are offset from each other for clarity.

We investigated the ability of an ensemble to reconstruct an input waveform in different noise regimes. In order to focus on stimulus reconstruction *via* ensemble event detection, we drove the Wang-Buzsaki model neuron with a frequency-modulated (FM) waveform. The FM waveform is simpler than a general Gaussian process, in that comprises a series of distinct upswings of equal amplitude, but at variable intervals and slopes. We compared the response at a bifurcation point under two circumstances: low noise and medium noise. In this way we could compare responses at the same amplitude with a similar, but not identical, spike rate. For the low-noise case in Figure 6A, there were six events during the time interval displayed (bottom, the ticks representing the spikes coalesced into gray vertical lines), but for the medium-noise case there were additional events with a reduced reliability and precision (top). We applied the event-based analysis to the entire simulation time interval (1100 ms) and determined for each event the reliability and precision (Figure 6B). We also determined the spike-triggered average (STA) of the stimulus for both cases (Figure 6C). Using all detected events with a reliability exceeding 5% and the measured STA, we reconstructed the stimulus waveform (Figure 6D). The ensemble of medium-noise spike trains, with their multiple spike patterns, yielded a better reconstruction (middle) than the low-noise case (top) from a single spike pattern.

To interpret these results, consider the response of a neuron to a volley with an average number *n* of spikes (*n = R×N*, where *R* is the event reliability and *N* is the number of neurons in the ensemble) each with some jitter. Each event contributes to a reconstruction of the input according to its reliability and precision. The gray shading in Figure 6B contains those events that might contribute to the reconstruction because they have the most reliable spikes with the lowest jitter. The shape of the shaded area represents the intuitive idea that when the precision is less, you need more input spikes to still produce an output spike, i.e. a higher reliability. The shown curve is hypothetical, but it is based on the simulation results reported in [49], [50], [51]. Experimental support derives from studies using two-photon uncaging of glutamate to produce synchronous ensembles of inputs that trigger dendritic action potentials and thereby reliably and precisely produce somatic action potentials [31].

The beneficial effects of the medium noise regime demonstrated for a frequency-modulated drive hold for more general drives, including the aperiodic drive used in the experimental studies. The basic requirement is that there are three noise regimes, which is the case for fluctuating drives, be it periodic or aperiodic, but their ranges in terms of the range of noise standard deviation vary with the specific details of the drive. For weak noise, the neuron spikes at perfect reliability and the jitter is proportional to the square root of the noise strength. The spikes sample only some of the upswings. For medium noise, there are still precise events, but the reliability is reduced, because there are multiple patterns. Due to the multiplicity of patterns more upswings are sampled. For strong noise, there are no longer discrete spiking events any more, rather the spike time density follows the temporal dynamics of the driving current. The beneficial effect occurs for medium noise strength, when more upswings are sampled, but the events are still precise enough to have a strong postsynaptic effect.

## Discussion

Previous studies have shown that neurons *in vitro* produce precise and reliable spike trains in response to fluctuating currents injected at the soma [52], [53], and neurons *in vivo* may also be capable of responding as precisely and reliably [6], [7], [14], [54]. We conducted *in vitro* experiments and computational simulations to address two related issues: First, to what extent are spike patterns conserved under common modes of response modulation such as current offset and fluctuation amplitude; Second, is there an optimal operating regime for representing this information in ensembles of uncoupled, similarly tuned neurons.

We found that precise spike times – whether events across trials or synchronous volleys in a neural ensemble driven by common input – are generated by rapid upswings in the fluctuating drive. The timing of peaks in the input current persisted across changes in current offset and amplitude and could therefore preserve information about the stimulus despite changes in offset and amplitude, gain and sensitivity, or neuromodulation and network activity [55]. However, the specific temporal pattern of spikes trains could change in a discrete way as these conditions varied. Event times typically changed little with changing amplitude or offset until a bifurcation was encountered, at which point they changed suddenly. Because of afterhyperpolarization currents and other slower currents, a sudden shift of one spike time led to changes in subsequent spike times for several hundred ms.

For a leaky integrate-and-fire neuron, jitter in spike times in response to a constant stimulus with noise is inversely proportional to the rate of change of the membrane potential at the threshold [56], which is in turn proportional to the current offset. For a fluctuating drive, the spike times are generated by upswings in the current drive, yielding a specific pattern of spike times. Usually there are more upswings than there are spikes produced by the neuron. Thus, when one of the upswings is missed, spikes are produced at a new sequence of upswings, and a new pattern appears. Nevertheless, the spikes in the new pattern still reflect some features of the stimulus waveform. Consider the case where one upswing brings the neuron close to threshold, without actually producing a spike. With a small increase in amplitude or current offset, the neuron will cross threshold, and a large change in the response will occur. This rapid change in the response for a small change in a parameter indicates the presence of a bifurcation. Such sensitivity to parameter changes also implies sensitivity to noise. On some trials the noise can induce a spike, whereas on others it prevents a spike, which means that in the presence of noise at least two patterns may be produced. Bifurcations are also present in nonlinear generalizations of the leaky integrate-and-fire neuron that may be more appropriate for cortical neurons [57]. We have explored the effect of adding a slow potassium current to the WB model (see Figures S2, S3). This current was described by a gating variable that was activated by action potentials, with a time constant of 10 ms and decayed to zero with a time constant of 500 ms for membrane potentials near rest. The dynamics in the presence of this current lead to bifurcations as a function of amplitude and offset, furthermore noise induced multiple patterns that could last for seconds.

Near bifurcation points, spike trains were more noise sensitive and the R-reliability was reduced. Noise here was the component of the neuron's input that varied from trial to trial. In the context of models this variability corresponded to an additional pseudorandom current, with a different seed on each trial [22]. In real neurons, channel noise and synaptic noise also contribute. Noise sensitivity is often considered detrimental to coding. At the ensemble level, however, it is beneficial because near bifurcation points multiple patterns are generated with high precision, each of which provides information about a different set of stimulus features. Furthermore, the overall spiking activity was still precise and informative because a range of spike patterns was obtained across a range of amplitudes and offsets, each representing upswings in the stimulus. Some neurons with low offset encoded only the largest and most salient features with a sparse pattern of spikes [58], [59], [60], while others with high offset revealed finer details in the stimulus at a higher firing rate [4]. In a recent study the response of a neuron in the barrel cortex of a rat was recorded while the rat moved its whiskers across a textured surface [61]. The neuron responded with highly precise spikes to stick-slip events on the surface, which manifested themselves as peaks in the whisker acceleration, but it responded only to a small fraction of them. Hence, each individual neuron responded sparsely but precisely to peaks in the acceleration. Our analysis suggests that an ensemble of such neurons at a bifurcation point would provide information about each acceleration peak.

Clearly, the temporal fluctuations of the injected current play a role in establishing the timing of possible spiking events. However, for any particular cell, the spike patterns emerge from the interplay of both the time-varying injected current and the cell's active currents and/or reset. Bifurcations are due to new spiking possibilities, when the membrane potential comes closer to threshold. Crossing threshold causes a spike, but because of the afterhyperpolarization (or other slow currents) it also changes the spike times that come after it, sometimes preventing a spike in favor of a new, later, spike time. This form of history dependence could thus lead to a new pattern (or patterns), assuming that such spiking opportunities continue to occur with greater frequency than the neuron's firing rate can keep up with, on average. When the neuron is close to a bifurcation, noise can switch between the two patterns (one below the bifurcation point and one above it); here “above” and “below” refer to the value of the bifurcation parameter. In summary, bifurcations are generated by the interaction between the temporal dynamics of the drive, the intrinsic time scales and the size of the reset, AHP, and other currents.

The bifurcation structure is determined by stimulus characteristics as well as the properties of the neuron. The effect of a slow potassium current presented earlier demonstrates the relevance of intrinsic properties. The role of the stimulus statistics is demonstrated by comparing the structure of the bifurcation obtained in response to a periodic drive with that for an aperiodic drive. For a periodic injected current, *I(t)* = *α+β* sin(ω*t*), with amplitude *β* as the variable parameter, the density of bifurcations is highly non-uniform. There are large parameter ranges without any bifurcations separated by a range full of bifurcations (see Figure 3A in [62]). When a bifurcation does occur, it causes changes in the spike trains for seconds. This has also been analyzed in experimental and simulation data by using interspike interval return maps [63], [64], [65]. In contrast, for an aperiodic drive applied over a long time interval, there is a high probability that a bifurcation will occur because a randomly selected drive will come arbitrarily close to threshold at some point during the stimulus presentation (see Figure 5B). The state of the neuron can often be reset by a large hyperpolarization or depolarization. Although long stationary episodes do occur in vivo, especially during sleep, during wakefulness resets occur in the visual system every time the eye moves, approximately 3 times a second. Thus the time interval over which the bifurcation structure is valid can vary over a wide range from tens of ms to tens of seconds. What impact do these bifurcation points have on cortical function?

### Implications for cortical coding

Our overall goal was to use the *in vitro* experiments to gain insights into the dynamics of neural ensembles *in vivo*. This raises two issues: First, what is the nature of neural ensembles, and second, how do they represent information?

There are two extreme hypotheses about how information is represented in an ensemble. The first holds that slow modulation (50–500 ms) of the firing rate encodes stimulus properties. This firing rate can be estimated by averaging across the relevant neurons in the ensemble, from which the time course of the stimulus can be reconstructed. The second hypothesis is that ensembles of neurons produce precisely timed spikes, which lead to synchronous volleys or events that are effective in driving postsynaptic neurons. Experimental results, reviewed in [14], suggest that a combination of these two strategies is at work at the level of the cortex. First, volleys represent the fast fluctuations in the inputs to the cortex [19] or internally generated events [6], [7]. Second, slow modulations change the number of volleys and the number of spikes per volley. The two information channels can work together on two different time scales and even interact. More spikes are fired in neurons whose tuning properties better match the sensory input, which raises the level of activity in the ensemble and engages new dynamical mechanisms, such as gamma oscillations, that in turn generate synchronous events within the cortex and increase downstream firing rates [66], [67].

Spatially clustered, temporally precise synaptic inputs to pyramidal neurons *in vitro* are effective at eliciting reliable and precise spikes through dendritic action potentials [31]. This decoding mechanism is decoupled from the spikes generated by slow input modulations and has an all-or-none character. If, for instance, 50 spikes in a 2 ms long interval are enough to elicit an output spike, then increasing the number of spikes or their precision will generally not increase the number of spikes produced in response to this volley. Any volley meeting the minimum requirement will elicit a spike and will be able to transmit information about the upswing that generated it. Hence, when there are multiple precise spike patterns, the neural ensemble provides more information than a single pattern, despite the reduced reliability at the single neuron level. This suggests that it is beneficial for the nervous system to keep ensembles close to bifurcation points so that they are most informative about the dynamics of their inputs.

### Ensembles of similar neurons are a theoretical construct

In general, a neural assembly refers to a group of cells sharing dynamically in functionally related activity. An assembly may or may not correspond to an anatomically distinguished set of cells such as a cortical column. In our simulations, the network is essentially feedforward; experimentally, synaptic transmission was blocked. In the systems we considered, therefore, correlations resulted from common inputs. However, our analyses did not assume any specific underlying architecture, and so would apply generally to any ensemble of cells. In a separate recent work, we have explored the reliability of a similar network with added feedforward inhibition, and established that the influence of inhibitory inputs was negligible when compared to the influence of feedforward excitatory drive and synchrony [19].

There is ample experimental evidence for the existence of functional neural ensembles. For example, fewer than 5% of the synaptic inputs to spiny stellate cells in layer 4 of the cat visual cortex are from thalamic relay neurons [18], [68]. Dual recordings from thalamic neurons and cortical neurons reveal a high degree of convergence [10], and analysis of these data show that as few as 5 thalamic cells with 5 synaptic contacts are sufficient to reliably elicit a spike [19]. Thus synchronous volleys of spikes from thalamocortical neurons insure that visual information enters the cortex despite background activity in both the thalamus and the cortex. As another example, recordings in hippocampus and cortex show coordinated reactivation of small assemblies during sleep and quiet awakeness [6], [7]. Similarly, recent *in vivo* recordings in rodent barrel cortex [69] show that under certain circumstances nearby neurons have correlated membrane potentials, indicating that they receive similar inputs. Furthermore, their spikes are preceded by large, sharp deflections of the membrane potential, indicating the presence of synchronized volleys. Nevertheless, even neurons in the same cortical column have diverse morphologies, different input conductance and other physiological differences. Our analysis shows that despite this heterogeneity, temporal information is robust across a range of parameter values and can be combined into precise volleys even across a moderately heterogeneous ensemble.

### How noise in the cortex can be beneficial

Bifurcation points allow noise to enrich the representation of stimulus features. This effect superficially resembles subthreshold stochastic resonance (SR), but is distinct from it. In traditional SR [70], [71], [72] noise can enhance signal detection by allowing a spiking neuron to reach threshold even when the applied stimulus remains below threshold. SR is effective when a subthreshold stimulus is close enough to threshold to allow noise facilitated spiking. Such a stimulus is *a fortiori* near a bifurcation point, because increasing either the current offset or the amplitude of the fluctuating input will bring it above threshold. In this sense SR may be viewed as a special case of a bifurcation point phenomenon analogous to that described here. Both SR and spike-time bifurcation phenomena involve a time varying deterministic input signal and a detection process. In classic SR both the mean input signal and the entire signal remain below threshold, while in our experiments the mean input signal was suprathreshold. This distinction may be readily appreciated in a simple threshold-crossing model such as a leaky integrate-and-fire (LIF) neuron driven by additive noise currents. For simplicity, suppose a LIF model cell with unit membrane time constant is driven by a combination of fixed (deterministic or “frozen noise”) and noisy input currents:
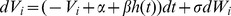
with the voltage reset to *V_0_ = 0* upon reaching the threshold *V_th_ = 1*. Here *dW_i_* represents white noise forcing (increments of a Wiener process), which is taken to be independent on each trial (or for each cell, in the simultaneous ensemble interpretation). The expression 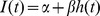 represents the injected current drive, with mean 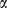, and *h(t)* a zero-mean, function with 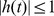, and a fluctuating component amplitude set by 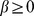. The function *h(t)* can either be a deterministic function, such as a sinusoid or combination of sinusoids, or a predetermined “frozen noise” stimulus. The detailed analysis of this system is a source of open mathematical problems [73]. The analysis of the system is qualitatively different in the suprathreshold case (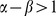), the subthreshold case (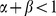) and the mixed or perithreshold case (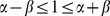) [74], [75]. If one were to assume the existence of a current threshold for spiking as in the LIF model, then in a similar way the applied stimulus (whether deterministic, such as a sinusoid, or stochastic, as in the frozen noise paradigm) may either be subthreshold (spikes cannot occur without noise), suprathreshold (spikes can occur at any time; spike occurrence is not noise dependent; noise only weakly modulates the times of individual spikes) or perithreshold (the stimulus repeatedly crosses the current threshold; spike occurrence and spike timing depend both on the noise and the stimulus; the hyperpolarizing downswings of the fluctuating component become large enough to exclude spiking over certain time intervals; this regime corresponds to the “forbidden zones” mechanism explored in [76]). In our preparation, the DC component of the input current (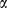, above) was always set to be large enough to guarantee that the cell would fire a train of action potentials. The mechanism of spike time precision and reliability in both the suprathreshold and perithreshold regimes is distinct from the mechanism underlying standard stochastic resonance in the subthreshold regime. The experiments reported here fall in the suprathreshold case when the stimulus amplitude is low, and transition to the perithreshold case as the stimulus amplitude 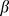 increases. The regime in which stochastic resonance may occur corresponds to the subthreshold case, which was not explored here.

Hunter and Milton demonstrated, in both *in vitro* and computer experiments, that spike timing reliability is sensitive to the frequency content of an aperiodic injected current in a way that depends on the relative amplitude of the fluctuating drive component [77]. For low amplitude inputs they found that noise dominated the spike times and that the reliability was low regardless of drive frequency content. For high amplitude inputs the spike times were determined by large current upswings, leading to high reliability regardless of frequency content. For intermediate amplitude inputs, however, the reliability was strongly influenced by input frequency content. Here we surveyed the entire range from weak amplitudes to strong. We found that bifurcation points enhanced the representation of inputs by neural ensembles primarily for intermediate amplitude values. This observation suggests that the presence of bifurcation points, and the frequency dependence of spike-time reliability [78], may occur in overlapping intermediate amplitude regimes.

The operating point of the cortex depends on many factors, including the level of arousal and the behavioral state of the animal. The presence of spontaneous background activity even during relaxed behavioral states suggests that the intracellular membrane potential is balanced just below threshold [79]. This allows neurons to react rapidly to sudden changes in synaptic inputs and also insures a high sensitivity to synchronous events in neural ensembles. Thus, the spontaneous activity should not be considered noise but a dynamical variable that can be adjusted in magnitude and variance to enhance the function of the cortical area. In particular adjusting the background activity could be one of the targets of some neuromodulators, such as acetylcholine.

### Limitations of the experimental study

The experimental results presented here apply to uncoupled neural ensembles receiving feedforward inputs. This clearly is an approximation to *in vivo* dynamics because: (1) There are recurrent synaptic connections that generate coherent activity in various frequency ranges and across different spatial scales [80]; (2) There is feedforward inhibition that follows feedforward excitatory volleys at a small delay [81] and (3) There are recurrent loops between the different cortical layers [82], [83], [84]. Nevertheless, at the soma/spike generating zone the sum of these inputs leads to a fluctuating drive. This fluctuating drive consists of fast fluctuations riding on top of slower modulations. We have shown that spike timing is generated by these fast fluctuations and modulated by slow fluctuations. The relative phase between gamma oscillations in two brain areas can modulate the effectiveness of communication [85]. Hence, an important issue for further study is how fast feedforward volleys interact with internally generated oscillations in the gamma frequency range [67].

In the *in vitro* experiments, current is injected at the soma. But *in vivo* the excitatory and inhibitory synaptic inputs are spatially distributed across the soma and dendritic tree. In particular, somatic current injection bypasses the nonlinear integration that takes places in the dendrites [86], [87]. In addition, the changes in conductance due to opening of synaptic channels are absent from the current injection. We have partially accounted for these dendritic effects by varying the amplitude and offset of the current injection motivated by analyses of the effects on the firing rate versus current (f-I) characteristic. Adding a constant conductance approximately shifts the f-I curve of a leaky integrate-and-fire neuron to the right, similar to the response of a hyperpolarizing current offset [88]. Synaptic inputs add a fluctuating conductance, which in models can change the gain of the f-I multiplicatively and increase the impact of other fluctuations. *In vitro* experiments show random background activity produced by a network can cause changes in the gain or sensitivity to other inputs [89], [90]. This is not the same as changing the amplitude, but the effects on the neuron are similar.

The offset and amplitude approximate a number of effects. First, because neurons in the ensemble are not identical, there is diversity of offset and amplitude values across the ensemble. Our results show that despite this diversity, the ensemble can produce synchronous volleys. Second, when the network state changes, as might occur in response to top-down modulation, the overall offset and amplitude changes. *In vivo* experiments have documented corresponding changes in gain and sensitivity in response to top-down activation of cortical networks [28], [91]. Interestingly, the *in vitro* experiment across trials may be a better approximation of a neural ensemble on one trial than the response of one *in vivo* neuron across multiple trials because the drive component that varies from trial to trial has a large shared component among different neurons in the ensemble [60].

### Analysis method

Precision and reliability are distinct properties of neural dynamics, although they may be modulated in a correlated fashion. There is a need both for an easy way to characterize the overall variability as well as for parsing out the reliability and precision separately. A reliability measure such as R-reliability [43] is appropriate for the former purpose, because it quantifies the overlap between pairs of spike trains, at a given temporal scale. But it does not directly indicate whether this overlap is due to a reduced reliability or precision, nor whether there are multiple patterns. Because reliability and precision are event properties, the event structure needs to be extracted in order to quantify them. Distinct events may be overlapping, however, making it difficult to separate them. As an example, consider sampling from two Gaussian densities with means of −0.5 and 0.5, respectively, both with a standard deviation of 1. It is not possible to say to which of the two distributions a sample point at 0 belongs to. However, by exploiting the context provided by the history of the spike train, the overlapping events can be separated. Hence the ensemble of spike patterns plays a crucial role in disentangling the reliability and precision of the spike train ensemble. Because the event-based analysis procedure only depends on a few well-defined parameters for which heuristics are available, it is reproducible from lab to lab [34]. Briefly, the temporal resolution at which the spike patterns can be optimally distinguished is first determined; then the number of patterns is found; after which the events for each pattern are determined and finally, events common to multiple patterns are merged.

### Future studies

Mathematically, bifurcations of spike time patterns due to addition or removal of individual spikes resemble so-called grazing bifurcations present in non-smooth dynamical systems such as impact oscillators [92], [93], [94], [95]. Recent advances in the understanding of stochastic suprathreshold leaky integrate and fire model neurons may help clarify the structure of spike time bifurcations in mathematical detail, although many details remain for future study [73].

Optogenetic technologies utilizing light-activated channels and pumps together with the read-out of neural activity via two-photon microscopy makes it possible to manipulate and record from neural ensembles *in vivo* [96], [97], [98], [99], [100]. This should make it possible to move neural ensembles away from or towards bifurcation points. Furthermore, *in vitro*, at the single neuron level, rapid spatially-distributed glutamate uncaging [31], [101] can be used to determine how neurons respond to the feedforward inputs generated by a neural ensemble and how these inputs interact with pharmacologically generated fast oscillations. Taken together, these technologies offer the opportunity to test *in vivo* predictions about the functional role of neural ensembles positioned at bifurcation points.

## Methods

### General experimental procedures

The voltage response of cortical neurons was measured in a rat slice preparation as described previously [102]. The Salk Institute Animal Care and Use Committee approved protocols for these experiments; the procedures conform to USDA regulations and NIH guidelines for humane care and use of laboratory animals. Briefly, coronal slices of rat pre-limbic and infra limbic areas of prefrontal cortex were obtained from 2 to 4 weeks old Sprague-Dawley rats. Rats were anesthetized with isoflurane and decapitated. Their brains were removed and cut into 350 µm thick slices on a Vibratome 1000 (EB Sciences, Agawam, Mass.). Slices were then transferred to a submerged chamber containing standard artificial cerebrospinal fluid (ACSF, mM: NaCl, 125; NaH_2_CO_3_, 25; D-glucose, 10; KCl, 2.5; CaCl_2_, 2; MgCl_2_, 1.3; NaH_2_PO_4_, 1.25) saturated with 95% O_2_/5% CO_2_, at room temperature. Whole cell patch clamp recordings were achieved using glass electrodes containing (4–10 MΩ; in mM: KmeSO_4_, 140; Hepes, 10; NaCl, 4; EGTA, 0.1; Mg-ATP, 4; Mg-GTP, 0.3; Phosphocreatine, 14). Patch-clamp was performed under visual control at 30–32 °C. In most experiments Lucifer Yellow (RBI, 0.4%) or Biocytin (Sigma, 0.5%) was added to the internal solution for morphological identification. In all experiments, synaptic transmission was blocked by D-2-amino-5-phosphonovaleric acid (D-APV; 50 µM), 6,7-dinitroquinoxaline-2,3,dione (DNQX;10 µM), and biccuculine methiodide (Bicc; 20 µM). All drugs were obtained from RBI or Sigma, freshly prepared in ACSF and bath applied. Data were acquired with Labview 5.0 and a PCI-16-E1 data acquisition board (National Instrument, Austin Tex.) at 10 kHz, and analyzed with MATLAB (The Mathworks).

### Stimulus generation and experimental design

We applied the event finding method to data collected to study the effect of varying the amplitude and offset of a repeated “frozen noise” stimulus. For all experiments the same frozen noise waveform *h(t)* was used. A white noise waveform (sampling rate 10 kHz, with samples uniformly distributed on the unit interval) was generated using the MATLAB function *rand* with the state of the random number generator set to zero. It was twice filtered using the MATLAB routine *filter(a_fil_,b_fil_)*. First, we applied a low-pass filter with a corner frequency of 100 Hz, obtained by setting *a_fil_* = [1, −0.99] and *b_fil_* = 1. Second, we performed a 50-sample (5 ms) running average (*a_fil_* = 1, *b_fil_* has fifty elements equal to 1/50). The first 500 samples (i.e. 50 ms) of the transient were discarded and the remaining waveform was centered on zero by subtracting the mean and normalized to have unit variance by dividing by the standard deviation. Depending on the cell and the quality of the seal, the waveform was multiplied by a factor ν representing the maximum amplitude. Waveforms with fractional amplitudes (*b*) ranging from zero to one were presented to the cell, as listed in the main text. In other experiments the waveform amplitude was held constant but its offset (*a*, i.e. mean) was varied instead. We also conducted experiments to determine the effect of the initial state of the neuron, which for our purposes was the membrane potential at the start of the actual stimulation, on the response to a fluctuating stimulus waveform. In these experiments, the level of depolarization of the initial constant-current step was varied, but the amplitude and offset of the fluctuating current was held constant. The fluctuating stimulus was increased in length to 1650 ms without changing the initial 1050 ms and a longer constant step was put in front of this stimulus.

Trials were separated by at least 15 sec of zero current injection, to let the membrane return to its resting state. Throughout the experiment, a few hyperpolarizing pulses were injected to monitor the access resistance of the preparation. These pulses were clearly separated from other stimuli.

### General analysis procedures

Spike times were detected from recorded voltage traces as the time the membrane potential crossed 0 mV from below. The firing rate was the number of spikes recorded during a trial, averaged across all similar trials and normalized by the duration of the trial in seconds. Here “similar” means having the same amplitude and offset.

In the rastergram, each row represented a spike train from a different trial. Each spike is represented as a tick or a dot, with the x-ordinate being the spike time and the y-ordinate being the trial number. Often we group trials together based on the stimulus amplitude or re-order trials based on which pattern they belong too. This is indicated in the corresponding figure caption.

The spike time histogram is an estimate for the time-varying firing rate. It was obtained by dividing the time range of a trial into bins (typically 0.5 ms wide) and counting the number of spikes that fell in each bin across all trials. The bin count was normalized by the number of trials and by the bin width in seconds. The latter was to assure that a bin entry had the dimensions of a firing rate, Hz. The histogram was subsequently smoothed by a Gaussian filter with a standard deviation equal to 4 bins. The spike-triggered average (STA) was obtained for each spike by selecting the 25 ms stimulus segment prior to the spike and averaging across all spikes.

Events were detected using the procedure summarized below. At the end of this procedure, all spikes were either assigned to an event or were classified as noise. The event-reliability is the fraction of trials on which a spike was observed during that event, and the event-jitter is the standard deviation of the spike times belonging to the event. The event-precision is the inverse of the event-jitter. For a given condition (amplitude, offset or initial current step) the reliability, precision and jitter are defined as the event-reliability, event-jitter and event-precision averaged across all events.

The R-reliability is calculated based on all spike times without detecting events. The spike trains are first transformed into a continuous waveform, where each spike is convolved with a Gaussian distribution with a standard deviation sigma [43], [103]. This procedure eliminates quantization noise that would otherwise result from *a priori* binning of the spike time data [104]. The cosine of the angle between the two waveforms, when considered as vectors, is computed as the inner product between the waveforms of trial *i* and *j*, normalized by the product of the square roots of the inner product of each trial with itself. This quantity is a number between 0 and 1 (the waveforms are positive valued) and is called the similarity *S_ij_*. The reliability estimate *R* is the mean of *S_ij_* across all distinct pairs <*ij*>. Intuitively, the inner product measures the degree of overlap between spike times: the closer two spike times are, the larger the overlap and thus their contribution to the inner product. Sigma sets the time scale of the reliability measure and determines which spike times between the pairs are considered overlapping. For sigma approaching zero, all spike times are considered different (except when the spike trains are identical to machine precision), hence *R* = 0. For sigma much larger than the trial length, all spikes overlap and *R* = 1. In the first case, the emphasis is on precise spike times; in the second case, the emphasis is on the global amount of spikes (spike rate). We used a more efficient method for calculating R by summing, for each pair of trials <*ij*>, the following expression across all spike pairs <*kl*> that are separated by less than six sigma's, 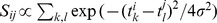 (here 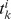 is the *k^th^* spike on the *i^th^* trial and for simplicity the normalization was omitted, see [104] for details.

### Calculation of the VP distance

Briefly, the Victor-Purpura (VP) metric [105] calculates the distance between two spike trains *A* and *B* by calculating the cost of transforming *A* into *B* (or *B* into *A* - the measure is symmetric). This distance is obtained as the minimum cost of transformation under the following rules: adding or removing a spike from *A* costs +1 point, while sliding spikes forward or backward in time by an interval *dt* costs *q* times 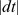. The variable *q* (units 1/ms) represents the sensitivity of the metric to the timing of spikes. For large *q* values it is frequently cheaper to add and remove spikes than to move them. Hence, for very large *q*, the metric is simply the number of spikes with different times between the two trains. For small *q* values, spike moving transformations are cheap, leaving the majority of the metric's value to the difference in the number of spikes which must be added or removed to produce train *B*; in the limit, the metric becomes the difference in the number of spikes in each spike train.

### Overview and goal of the event finding method

In a recent study we uncovered multiple spike patterns in trials obtained in response to repeated presentation of the same stimulus both *in vitro* and *in vivo* [4], [54]. Spike patterns are present when trials, or at least short segments thereof, can be separated in two (or more) distinct groups of spike sequences. As an example consider the case where on some trials the neuron spikes at 10 and 35 ms (relative to stimulus onset), whereas on other trials it spikes at 15 and 30 ms. These trials would consist of two distinct patterns as long as there are no trials with spikes at 15 and 35 ms or at 10 and 30 ms. Hence, spike patterns correspond to a within-trial correlation, because, in the example, a spike at 10 ms implies that a spike will be found at 35 ms with a high probability. The problem of finding spike patterns is made harder by the presence of spike time jitter and trial-to-trial unreliability.

We designed a method to uncover patterns independently of the event structure and then used the patterns to construct the event structure. The full method is described elsewhere [34], here we only summarize the basic steps of the method. The method itself is unsupervised, but four parameters need to be provided. The four parameters are: the threshold for finding events (parameter *t_ISI_*); the temporal resolution for which two spike times are considered similar (parameter: *q*); and the number of patterns the clustering algorithm looks for (parameter *N_c_*, which stands for the number of clusters); and a threshold *t_ROC_* which determines which events need to be merged because they are common to multiple spike patterns. For the parameter settings used here, each cluster corresponds to a spike pattern, hence we will use these designations interchangeably. The method has been tested on short temporal segments with on average approximately 2 or 3 spikes per trial. These segments can be found by cutting spike trains at times with a low or zero spike rate in the spike time histogram. In the following text, we assume that the data has been divided in segments and discuss the analysis on one segment.

The basic premise is that two trials on which the same pattern was produced are more similar to each other than two trials on which different patterns were produced. We used the VP distance [105] to quantify the similarity. The distance between spike trains *i* and *j* is represented as a matrix *d_ij_*. The distance matrix appeared typically unstructured when the trials were arranged in the order in which they were recorded. We seek to re-order the matrix so that it becomes block-diagonal. The block diagonals correspond to trials that have a mutually small distance between all pairs in the block and are more distant to trials outside the block. That is, blocks on the diagonal correspond to spike patterns. This goal is achieved using the fuzzy c-means (FCM) method [106] applied to the columns of the distance matrix. FCM finds *N_c_* clusters and assigns to each trial a probability of belonging to a cluster. If the clustering is ‘good’ each trial belongs to only one cluster with a high probability, it is ‘poor’ if a trial has similar probabilities of belonging to two or more different clusters. We have developed a heuristic to find appropriate values for *N_c_* based on the gap-statistic [107], [108]. Once patterns have been uncovered, a preliminary event structure is determined using the interval method outlined in [64] *on each pattern*. The interval method operates on the aggregate spike train, comprised of the time-ordered set of all spikes across all trials. The interval method is based on the principle that in the aggregate spike train the distance between spikes within an event is less than the distance between spikes in different events. The common events that occur in multiple spike patterns were found and merged based on a receiver operating characteristic (ROC) analysis [109], which quantified how distinguishable the spikes in the two events were. After this analysis a cluster-assisted event structure is available. The result is illustrated in Figure 3B: each trial is assigned to a pattern on each segment, and each spike is assigned to an event. See [34] for additional examples.

### Calculation of entropy and mutual information between classifications

The outcome of the pattern-finding (clustering) procedure is that each spike train is assigned to a cluster. Formally, a set of trials has a classification *c_i_*, where *i* is the trial index between 1 and *N_trial_*; and the classification *c* is a number between 1 and the number of clusters *N_c_*. The class distribution *p_j_* is the fraction of trials that were classified as class *j*,
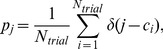
(1)where 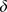 denotes the Kronecker delta. The diversity of the classification was characterized by the entropy
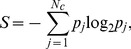
(2)where the sum was over all nonzero *p_j_* values because 0 log 0 was defined to be zero [110]. The entropy *S* was zero (minimal diversity) when all trials were assigned to the same class and was maximal at *S = log_2_N_c_* when all classes had the same probability of occurring (maximal diversity). It is well known that the estimation of entropy from histogram data is biased [111], [112]. We obtained approximate estimates for the bias and variance of the entropy estimates using a resampling procedure. Using the probabilities estimated from a finite number of trials as the exact probabilities we generated a thousand data sets from this probability distribution with the same number of trials and determined the entropy for each data set. The bias was the difference between the mean across the resampled entropy values and the original entropy, the variance was the standard deviation across the resampled entropy values.

The mutual information was used to measure the similarity between two classifications. The joint distribution between two classifications *c_i_* and *d_j_* with *N_c_* and *N_d_* classes, respectively, was computed as:
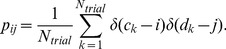
(3)The mutual information was then expressed as
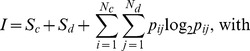
(4)

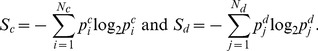
(5)


In the above formulas, the class distributions for *c* and *d* have a different subscript in order to distinguish them, hence *i* also is a class index rather than a trial index. To obtain a measure between 0 and 1 we normalized the mutual information by the maximum entropy, yielding the normalized mutual information:
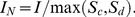
(6)


### Simulation experiments

The neuron was modeled as a single compartment with Hodgkin-Huxley-type voltage-gated sodium and potassium currents and a passive leak current [47], [113]. The equation for the membrane potential of the model neuron is:
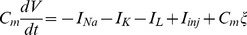
(7)where 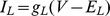 is the leak current, 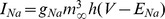 is the sodium current, 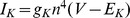 is the potassium current, *I_inj_* is the injected fluctuating current, which is the same on each trial and 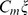 is a noise current that is different on each trial. The values for the maximum conductance and reversal potential are listed in Table 1. The gating variables are *m*, *n*, and *h* and they satisfy the equation
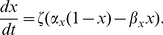
(8)Here the label *x* stands for the kinetic variable, and ζ = 5 is a dimensionless time scale that can be used to tune the temperature dependent speed with which the channels open or close. The rate constants are:
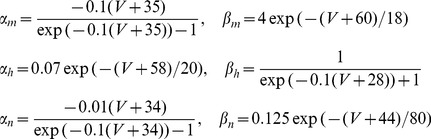
(9)and the asymptotic values of the gating variables are:
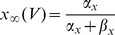
(10)where *x* stands for *m*, *n*, and *h*.

**Table 1:** Standard parameter values for the model neuron.

Parameter (units)	Value
*E_L_* (mV)	−65
*E_Na_* (mV)	55
*E_K_* (mV)	−90
*g_L_* (mS/cm^2^)	0.1
*g_Na_* (mS/cm^2^)	35
*g_K_* (mS/cm^2^)	9
*C_m_* (µF/cm^2^)	1

We made the approximation that *m* follows the asymptotic value *m*_∞_(*V*) instantaneously [47]. The noise *ξ_i_* in the current of neuron *i* is chosen such that 〈*ξ_i_*(*t*)〉 = 0 and 〈*ξ_i_*(*t*)*ξ_j_*(*t′*)〉 = 2*λδ*(*t−t′*)*δ_ij_*. On each integration time step, the noise was drawn independently from a uniform distribution between −12*λ*/*dt* and 12*λ*/*dt*, where *dt* was the time step. The random noise value was treated as a constant for the duration of the time step. For Figure 5 we used *λ = 0.00025* mV^2^/ms, whereas in Figure 6 we used *λ = 0.0001* mV^2^/ms (low noise) and *λ = 0.025* mV^2^/ms (medium noise). For *I_inj_* we either used the same 1050 ms long fluctuating drive as in experiment (Figure 5, amplitude between 0 and 1, offset 0.2 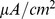), but without the constant depolarizing current pulses preceding the fluctuating drive *in vitro*; or we used a sinusoidal drive with time-varying frequency (illustrated in Figure 6).

For Figures S2 and S3 we performed simulations with an additional potassium channel added to the WB model. The gating variable was denoted by z and satisfied the following equation 
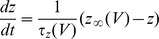
 with 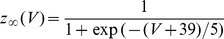 and 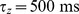 when 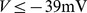 and 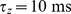 for higher membrane potential values. This resulted in an additional current 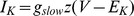 on the right-hand side of Eq. (7), with g_slow_ = 0.5 mS/cm^2^.

Additional parameters, Figure S2: offset 0.6 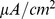, amplitude 0.4, *λ = 0* mV^2^/ms; Figure S3A: as 2, initial z-value as shown on the y-axis; Figure S3B: as 2, offset as shown on the y-axis; Figure S3C offset as shown on the y-axis and *λ = 0.01* mV^2^/ms.

The initial values of the membrane potential at the beginning of the simulation were set to a fixed value, usually −70 mV. The gating variables were set to their asymptotic stationary values, 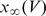, corresponding to the starting value, V, of the membrane potential. The differential equations were integrated using a second-order Runge-Kutta method with a time step of *dt* = 0.05 ms [114], [115].

## Supporting Information

Figure S1 **Additional examples of patterns correlated across the stimulus duration.** Each of six panels (A–F) has the same organization. The left and middle graphs are rastergrams, as in Figures 4A and 4B, respectively. The right-most panel displays voltage traces. The time interval was divided into 4 segments, which are indicated by thick vertical lines in the rastergrams. In the left most rastergram, spike patterns are determined in each segment separately, and each pattern is separated by a dashed horizontal line. In the middle rastergram, the trials are sorted according to the patterns in the fourth segment. In the right-most graphs, the bottom panel contains the driving current together with the spike rasters; the graphs above contain the mean voltage for each pattern (each depicted in a separate box) and the standard deviation is indicated by a gray band. Panels A and B, D, E and F, are examples where non-stationarity is visible as a periodic modulation in the spike times within the ellipses. We only placed one ellipse per panel, although there are more signs of non-stationarity in each of these panels. For panel C, the data is stationary and independent of initial condition. This visual assessment is borne out by determining the mutual information between the trial number and the pattern that is expressed in the fourth interval. For panels A to F it is (normalized mutual information between trial index and pattern, bias from resampling, standard deviation from resampling) A (0.1550, 0.0435, 0.0048), B (0.2384, −0.0820, 0.0042), C (0.0667, −0.0030, 0.0075), D (0.1857, −0.0567,0.0062), E (0.2805,−0.0914, 0.0062), F (0.1531, −0.0370, 0.0103). Note the normalized mutual information between trial index and pattern for panel C (0.0667) is significantly lower than that for the other panels, which range from 0.1531 to 0.2805.
(PDF)

Figure S2 **Slow currents generate long-lasting patterns.** We show the (A,B) voltage traces and (C,D) value of the gating variable z of the slow current as a function of time. Panels B and D are a close-up of C and D, respectively. There are 10 traces, each corresponding to a different initial z value (visible as different starting points at t = 0 in panel C). Because the first spikes occur at two different time points, two patterns emerge at the end of the trial, as indicated by the asterisk and the arrow. These patterns correspond to different voltage trajectories in panel B. However, during the interval depicted, the trials are still separating into patterns: the solid black curve does not show a spike just before 750 ms, but also does not spike at 850 ms, therefore the trajectory will merge to the “arrow” pattern even though it was not part of it at t = 750 ms. We used the Wang-Buzsaki neuron with an additional potassium current (strength 0.5 mS/cm2) with a gating variable z. The gating variable decayed to zero during rest with a time constant of 500 ms and charged up to 1 with a time constant of 10 ms during an action potential. See Methods and Experimental procedures for additional model parameters.
(PDF)

Figure S3 **Bifurcation structure in the presence of slow currents.** (A) Spike trains for different initial values of z. Approximately two patterns are reached. (B) Spike trains as a function of depolarizing current, bifurcations still occur and (C) represent sites of enhanced noise sensitivity. See Methods and Experimental procedures for model parameters.
(PDF)
