# Supplementary figures and images for: Multiple Spike Time Patterns Occur at Bifurcation Points of Membrane Potential Dynamics
Source: PLoS Comput Biol. 2012 Oct 18;8(10):e1002615. doi: 10.1371/journal.pcbi.1002615 (PMC3475656; doi:10.1371/journal.pcbi.1002615)

A

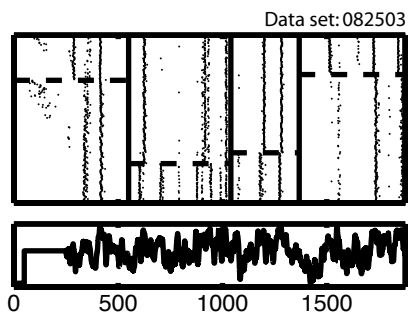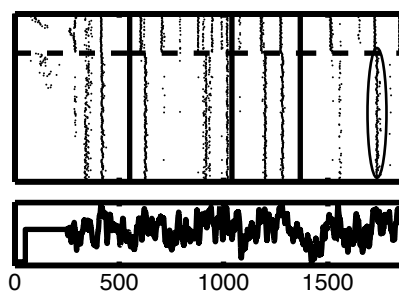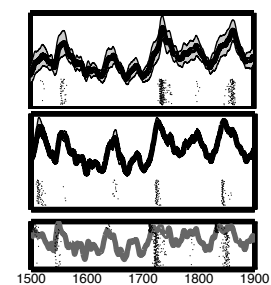

B

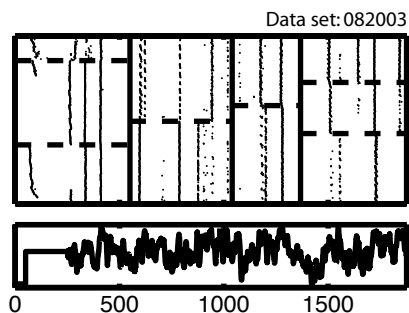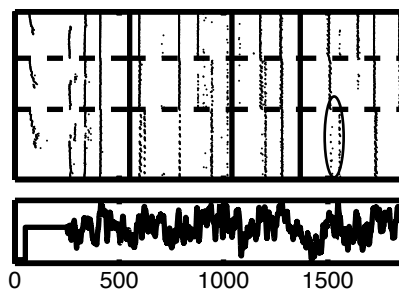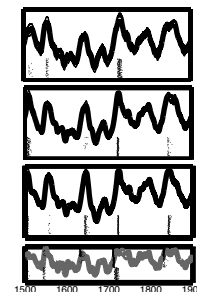

C

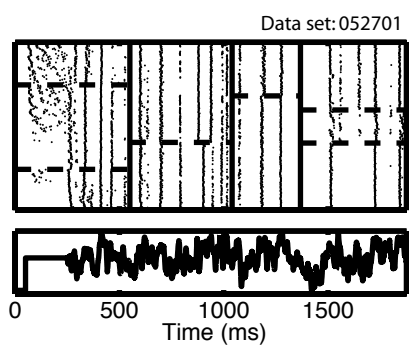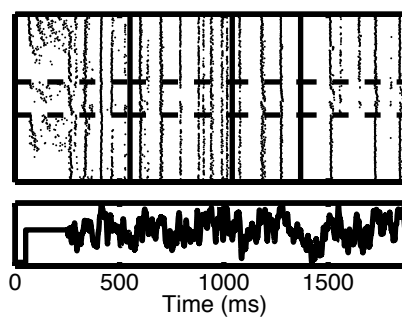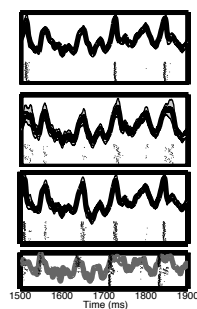

D

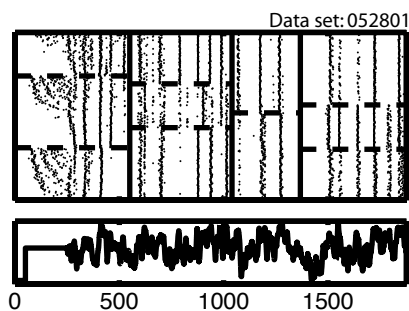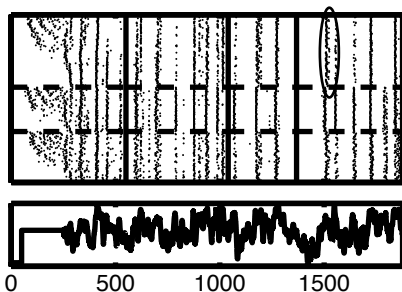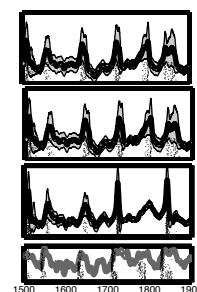

E

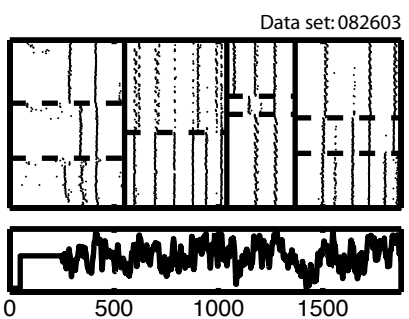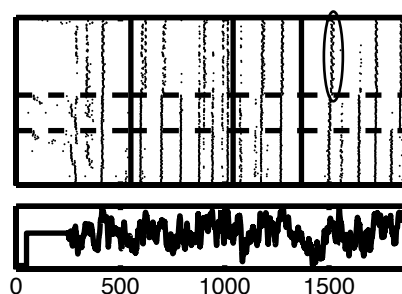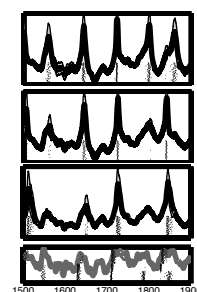

F

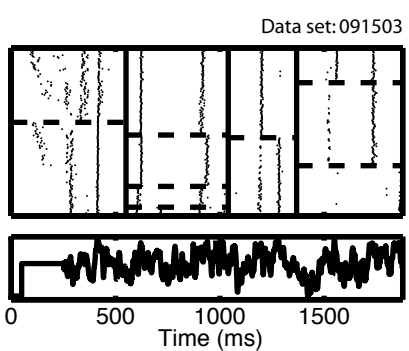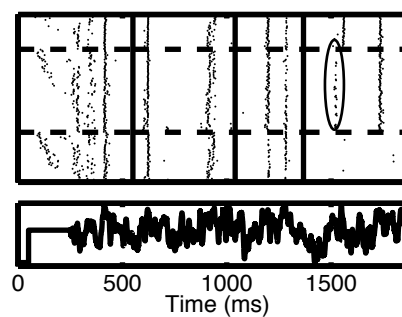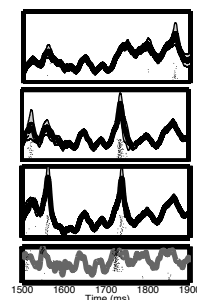

Supplement: Figure S1 — Additional examples of patterns correlated across the stimulus duration. Each of six panels (A–F) has the same organization. The left and middle graphs are rastergrams, as in Figures 4A and 4B, respectively. The right-most panel displays voltage traces. The time interval was divided into 4 segments, which are indicated by thick vertical lines in the rastergrams. In the left most rastergram, spike patterns are determined in each segment separately, and each pattern is separated by a dashed horizontal line. In the middle rastergram, the trials are sorted according to the patterns in the fourth segment. In the right-most graphs, the bottom panel contains the driving current together with the spike rasters; the graphs above contain the mean voltage for each pattern (each depicted in a separate box) and the standard deviation is indicated by a gray band. Panels A and B, D, E and F, are examples where non-stationarity is visible as a periodic modulation in the spike times within the ellipses. We only placed one ellipse per panel, although there are more signs of non-stationarity in each of these panels. For panel C, the data is stationary and independent of initial condition. This visual assessment is borne out by determining the mutual information between the trial number and the pattern that is expressed in the fourth interval. For panels A to F it is (normalized mutual information between trial index and pattern, bias from resampling, standard deviation from resampling) A (0.1550, 0.0435, 0.0048), B (0.2384, −0.0820, 0.0042), C (0.0667, −0.0030, 0.0075), D (0.1857, −0.0567,0.0062), E (0.2805,−0.0914, 0.0062), F (0.1531, −0.0370, 0.0103). Note the normalized mutual information between trial index and pattern for panel C (0.0667) is significantly lower than that for the other panels, which range from 0.1531 to 0.2805. (PDF) [file pcbi.1002615.s001.pdf]

A

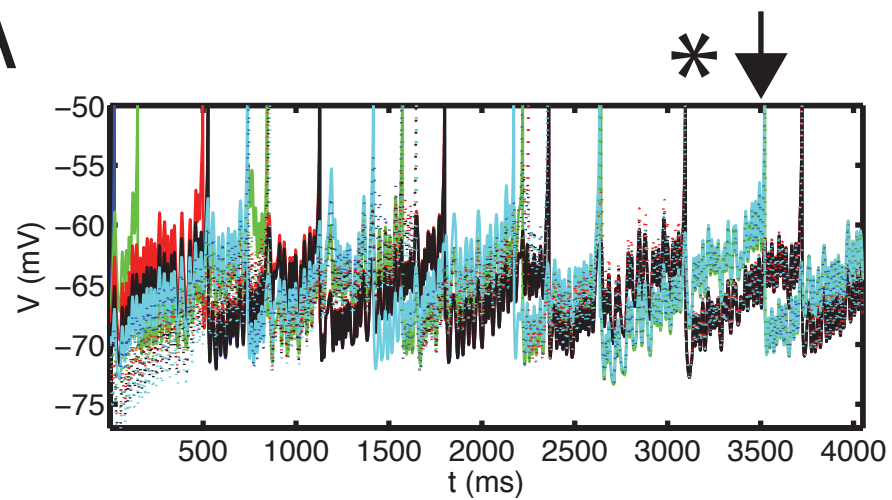

B

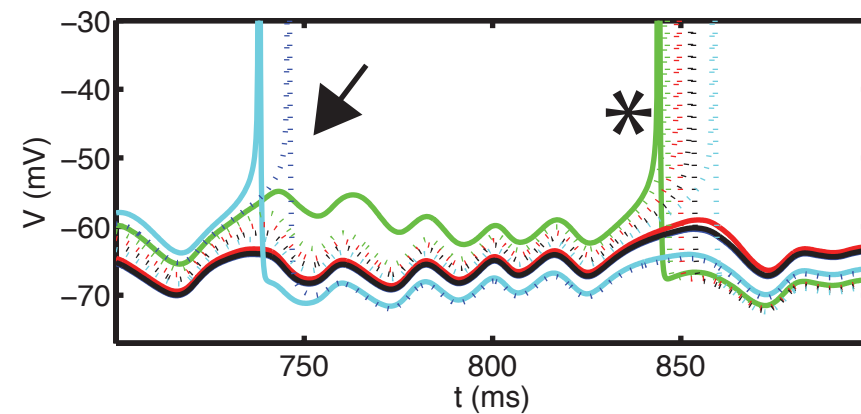

C

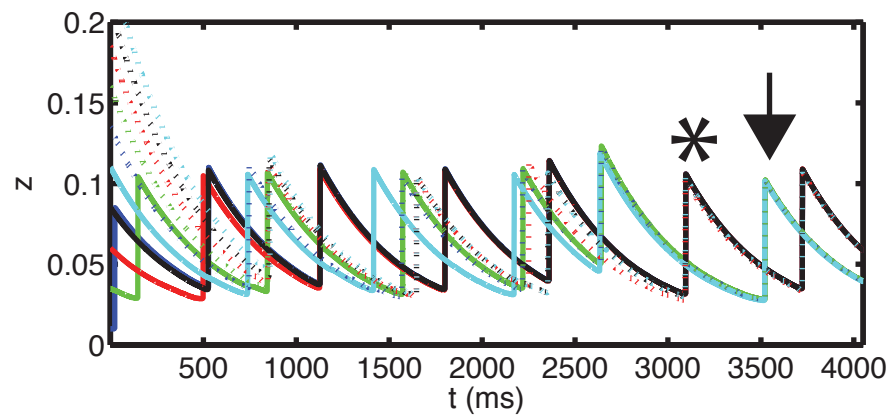

D

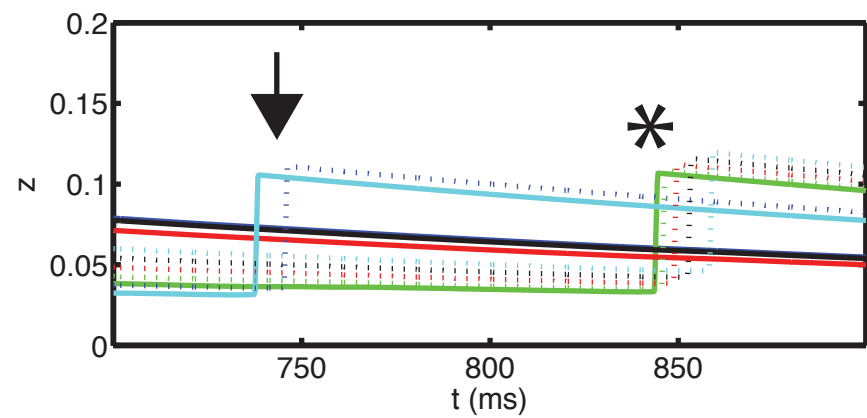

Supplement: Figure S2 — Slow currents generate long-lasting patterns. We show the (A,B) voltage traces and (C,D) value of the gating variable z of the slow current as a function of time. Panels B and D are a close-up of C and D, respectively. There are 10 traces, each corresponding to a different initial z value (visible as different starting points at t = 0 in panel C). Because the first spikes occur at two different time points, two patterns emerge at the end of the trial, as indicated by the asterisk and the arrow. These patterns correspond to different voltage trajectories in panel B. However, during the interval depicted, the trials are still separating into patterns: the solid black curve does not show a spike just before 750 ms, but also does not spike at 850 ms, therefore the trajectory will merge to the “arrow” pattern even though it was not part of it at t = 750 ms. We used the Wang-Buzsaki neuron with an additional potassium current (strength 0.5 mS/cm2) with a gating variable z. The gating variable decayed to zero during rest with a time constant of 500 ms and charged up to 1 with a time constant of 10 ms during an action potential. See Methods and Experimental procedures for additional model parameters. (PDF) [file pcbi.1002615.s002.pdf]

**A**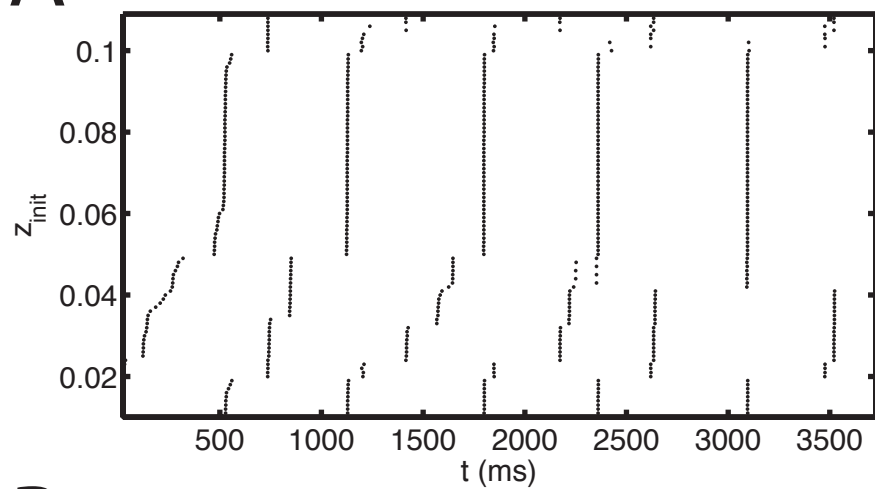**B**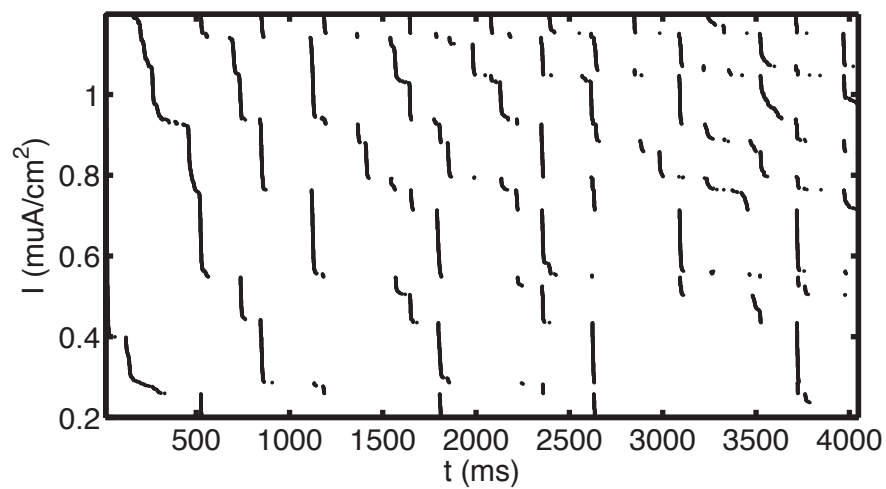**C**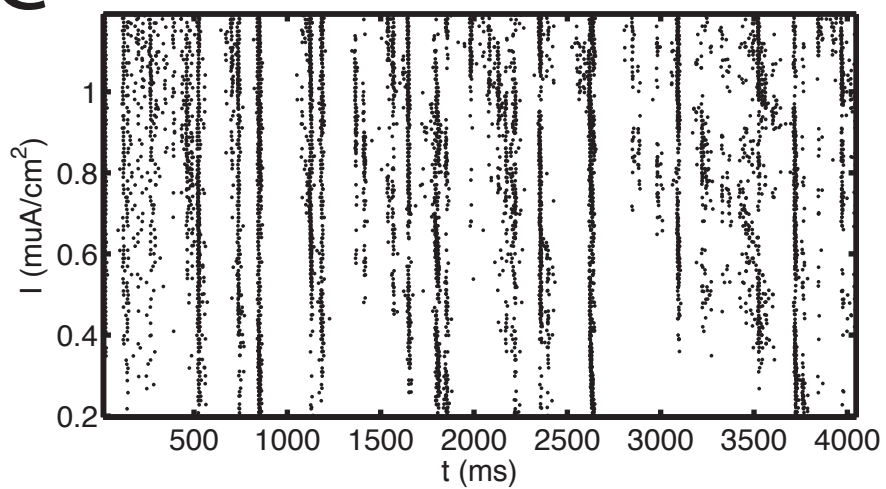

Supplement: Figure S3 — Bifurcation structure in the presence of slow currents. (A) Spike trains for different initial values of z. Approximately two patterns are reached. (B) Spike trains as a function of depolarizing current, bifurcations still occur and (C) represent sites of enhanced noise sensitivity. See Methods and Experimental procedures for model parameters. (PDF) [file pcbi.1002615.s003.pdf]
